# On-surface light-induced generation of higher acenes and elucidation of their open-shell character

**DOI:** 10.1038/s41467-019-08650-y

**Published:** 2019-02-20

**Authors:** José I. Urgel, Shantanu Mishra, Hironobu Hayashi, Jan Wilhelm, Carlo A. Pignedoli, Marco Di Giovannantonio, Roland Widmer, Masataka Yamashita, Nao Hieda, Pascal Ruffieux, Hiroko Yamada, Roman Fasel

**Affiliations:** 1 Empa, Swiss Federal Laboratories for Material Science and Technology, 8600 Dübendorf, Switzerland; 2 Graduate School of Materials Science, Nara Institute of Science and Technology (NAIST), 8916-5 Takayama-cho, Ikoma, 630-0192 Japan; 3 Department of Chemistry, University of Zurich, 8057 Zurich, Switzerland; 4 Department of Chemistry and Biochemistry, University of Bern, Freiestrasse 3, 3012 Bern, Switzerland

## Abstract

Acenes are an important class of polycyclic aromatic hydrocarbons which have recently gained exceptional attention due to their potential as functional organic semiconductors. Fundamentally, they are important systems to study the convergence of physico-chemical properties of all-carbon *sp*^2^-frameworks in the one-dimensional limit; and by virtue of having a zigzag edge topology they also provide a fertile playground to explore magnetism in graphenic nanostructures. The study of larger acenes is thus imperative from both a fundamental and applied perspective, but their synthesis via traditional solution-chemistry route is hindered by their poor solubility and high reactivity. Here, we demonstrate the on-surface formation of heptacene and nonacene, via visible-light-induced photo-dissociation of α-bisdiketone precursors on an Au(111) substrate under ultra-high vacuum conditions. Through combined scanning tunneling microscopy/spectroscopy and non-contact atomic force microscopy investigations, together with state-of-the-art first principles calculations, we provide insight into the chemical and electronic structure of these elusive compounds.

## Abstract (web summary)

The synthesis of large acenes via traditional solution-chemistry routes is hindered by their poor solubility and high reactivity under ambient conditions. Here, the authors demonstrate the on-surface formation of large acenes, via visible-light-induced photo-dissociation of α-bisdiketone molecular precursors on an Au(111) substrate.

## Introduction

Polycyclic aromatic hydrocarbons (PAHs), organic compounds comprised of two or more carbocyclic rings fused in linear, cluster, or angular arrangement, have gained increasing attention in fields, such as structural organic chemistry and material science^1,2^. Over the last decades, particular interest has been directed toward acenes, which constitute the family of PAHs consisting of linearly fused benzene rings. They have been the subject of intensive studies from both experimental and theoretical points of view due to their intriguing optical and electronic properties associated with their π-bond topology. One of the most appealing aspects in this regard is their extraordinarily narrow HOMO-LUMO gap in comparison with other PAHs with the same number of aromatic rings, which makes them promising candidates as novel semiconducting materials^3–5^. The nature of the electronic ground state of acenes has been widely debated in literature. While it is generally accepted that short acenes (up to pentacene or hexacene) have a closed-shell electronic structure, the ground state of longer acenes is still controversial. While early studies have predicted open-shell singlet^6^, triplet^7^ and even higher ground states (e.g. quintet^8^) for various acenes with *m* > 6 (*m* = number of benzene rings), recent studies have indicated an open-shell singlet ground state for longer acenes^9–11^. These studies also showed that this trend is accompanied by decreasing singlet-triplet and electronic gaps, significant rise in chemical reactivity, and increasing difference between ladder and rung carbon-carbon bonds, structurally rendering longer acenes as weakly coupled oligoacetylene chains. Furthermore, acenes can be viewed as the narrowest members in the family of zig-zag graphene nanoribbons (ZGNRs), which are predicted to host spin-polarized edge states^12,13^ and are postulated to be an important component in carbon-based spintronic devices.

However, the low solubility and high reactivity of acenes longer than pentacene has hindered their synthesis and characterization, with dimerization and photooxidation by molecular oxygen as typical degradation pathways^14–16^. Consequently, successful experimental investigations on such compounds are limited. In the last century, significant efforts were devoted towards the synthesis of pentacene^17^ and hexacene^18,19^, and only recently Bettinger and co-workers, in a pioneering work, have reported the formation of heptacene in the solid state after thermal cycloreversion from diheptacenes^20^. A recurrent strategy in the synthesis of larger acenes has been their functionalization with stabilizing and protecting groups^21–23^. In particular, hexacene was successfully isolated using monoketone precursors, and the structure was determined by single crystal X-ray diffraction^23^. Furthermore, the use of the Strating-Zwanenburg reaction^24^, where bridged *α*-diketone groups in acene-derived photoprecursor molecules undergo photodecarbonylation, has emerged as a promising approach for the synthesis of larger acenes. In fact, since the first report of using the Strating-Zwanenburg reaction^25^, synthesis of hexacene to undecacene (with the exception of decacene) has been successfully demonstrated, though the compounds needed to be stabilized in a PMMA matrix (hexacene and heptacene)^26,27^, argon matrix (octacene and nonacene)^28^ and a polystyrene matrix (undecacene)^29^, which kept them from decomposing for a few hours.

In view of these limitations, conventional solution synthesis has been extended to single-crystal surfaces under ultra-high vacuum (UHV) conditions, which provide a versatile route toward the formation of stable molecules. On-surface generation of molecular species also allows their direct structural and electronic characterization via advanced scanning probe techniques, such as scanning tunneling microscopy (STM) and non-contact atomic force microscopy (nc-AFM), together with complementary surface analysis techniques. Such techniques also serve as powerful tools to undertake a sequential study of various reaction steps, providing mechanistic insights on the synthetic pathway toward desired products. Established chemical reactions towards on-surface generation of tailored molecules, such as cyclodehydrogenation^30–32^, aryl-aryl coupling^30,33^ or covalent organometallic bond formation^34^—although greatly successful techniques—represent only a narrow spectrum of the on-surface chemistry toolbox. Contemporarily, acenes and their derivatives have been extensively investigated on metallic single crystals^35–37^ and insulating films^37–39^, though studies on larger acenes remain scarce^35,40^. Only recently, heptacene to undecacene^41–44^ have been synthesized on metallic substrates. Typically, the aforementioned on-surface reactions are triggered by a thermal annealing step relying on the catalytic role of an underlying metal substrate. However, light, like temperature, is also able to trigger on-surface chemical reactions providing novel photoexcited charge-transfer channels^45,46^, though its use is not as established as its thermally-driven counterpart. In this regard, while several studies of molecular photochemical on-surface reactions on single-crystalline metal substrates using UV-light have been reported^47–49^, only a few investigations on visible-light-induced reactions have been mentioned to date^50,51^. Thus, further investigations regarding light-driven on-surface reactions are imperative in order to access mechanistic insights.

In this article, we introduce an effective approach toward the on-surface formation of large acenes via photodecarbonylation of α-bisdiketone precursors on a coinage metal surface under UHV conditions. The establishment of on-surface large acene formation by photo-irradiation contributes not only toward developing the on-surface chemistry toolbox, but also provides experimental insights into the physical properties of large acenes. Our molecular-level investigations describe the visible-light-induced deprotection of heptacene and nonacene precursors, allowing their detailed structural characterization on an atomically clean and defined Au(111) substrate. In addition to our detailed chemical and structural investigation, we perform scanning tunneling spectroscopy (STS) measurements to characterize the electronic structure of heptacene and nonacene. In particular, we visualize the positive and negative ion resonances (PIR and NIR) that derive from the highest occupied and the lowest unoccupied molecular orbitals (HOMO and LUMO), and quantitatively extract the experimental electronic gaps. In combination with complimentary theoretical calculations, this allows us to rationalize the nature of the ground state of larger acenes (i.e. open- or closed-shell).

## Results

### Light-induced generation of heptacene and nonacene

**Fig. 1:**
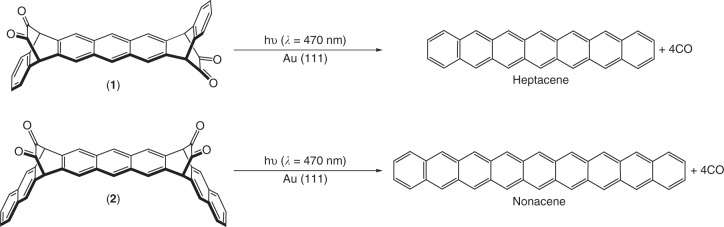
Light-induced on-surface formation of heptacene and nonacene. Schematic representation of the photodissociation of α-bisdiketone protected heptacene (**1**, top panel) and nonacene (**2**, bottom panel) precursors on a Au(111) surface to give rise to the formation of heptacene and nonacene, respectively

The synthesis of heptacene and nonacene precursors was accomplished by modified schemes of previous reports^28,40^ (see Supplementary Fig. 1–8 for synthetic details and characterization). Briefly, two α-diketone moieties were introduced to the backbone of large acenes as protecting groups to ensure stability and solubility. Importantly, the Swern oxidation was used in the final step to synthesize the nonacene precursor, with a significantly increased reaction yield^28^. Note that the *syn* and the *anti* conformers, present due to the introduction of the two α-diketone groups, were adequately separated during the purification. Figure 1 represents the expected on-surface reactions toward the formation of large acene species, which were previously reported in solution chemistry under related conditions^27,28^. Upon irradiation with visible light (wavelength (*λ*_1_) = 470 nm) the photodecarbonylation of α-bisdiketone heptacene (**1**) and α-bisdiketone nonacene (**2**) precursors is achieved giving rise to the formation of heptacene and nonacene, respectively. We chose a wavelength of 470 nm for precursor deprotection because it is close to both the *n*-π* transition of the diketone group of the precursor molecules (see Supplementary Fig. 9 for the UV-V is absorption spectrum of the α-bisdiketone heptacene precursor in toluene), and to the first interband transition of gold^52^, which may allow an efficient excitation of the Au(111) surface.

**Fig. 2:**
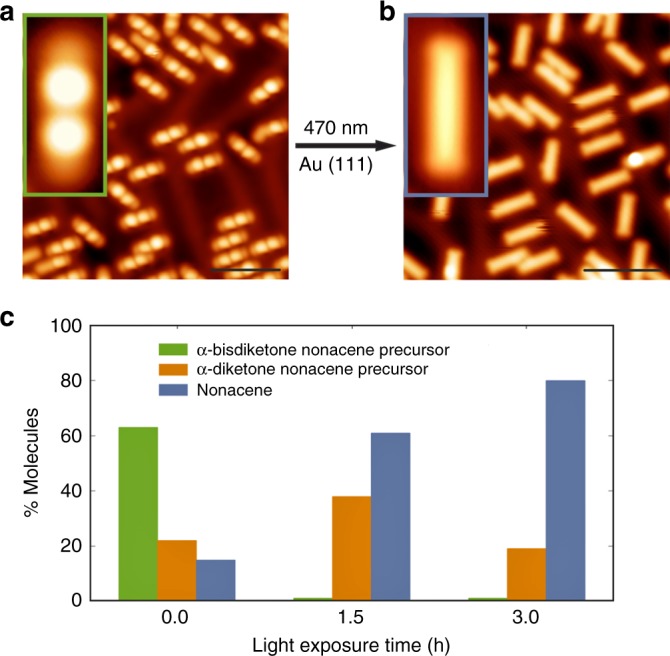
Nonacene formation via photodissociation of **2**. **a** STM image after room temperature deposition of the α-bisdiketone nonacene precursor on Au(111) surface. *V*_b_ = 0.7 V, *I* = 2 pA. Scale bar: 5 nm. The inset shows a high-resolution STM image of an individual α-bisdiketone nonacene precursor where two bright protrusions of identical apparent height are assigned to the upward-pointing bridged α- diketone groups. Image size 1.5 nm x 3.5 nm, *V*_b_ = −1.5 V, *I* = 4 pA. **b** STM image of nonacene species after light exposure (λ = 470 nm, 3 h) of the sample in panel **a**. *V*_b_ = −1 V, *I* = 7 pA. Scale bar: 5 nm. The inset shows a high-resolution STM image of an individual nonacene. Image size 1.4 nm × 3.4 nm, *V*_b_ = −1 V, *I* = 5 pA. **c** Graph depicting the percentage of photoconverted molecules vs. light exposure time. Statistics out of ~1100 molecules

Figure 2 illustrates the on-surface photogeneration of nonacene from **2**. Herein, a submonolayer coverage of **2** was sublimated under dark conditions on an Au(111) substrate held at room temperature. Figure 2a shows a constant-current STM image of mostly intact *syn* α-bisdiketone nonacene precursors (63% of the molecular species), exhibiting a rod-like appearance, and aligned in specific directions with respect to the surface. High-resolution STM imaging (inset in Fig. 2a) allows to discern the intramolecular features of **2**, wherein the two bright protrusions of identical apparent height (2.6 Å) are assigned to the bridged α-diketone groups pointing upwards (see Fig. 2c for complete statistics regarding the conversion process, and Supplementary Fig. 10 for density functional theory (DFT) calculations and simulated STM images of α-bisdiketone and α-diketone nonacene species adsorbed on Au(111)). Subsequently, we exposed the sample to visible light (*λ*_1_ = 470 nm, *ϕ*_photon_ = 2.5 × 10^18^ cm^−2^ s^−1^, *t* = 3 h). Figure 2b reveals that after light exposure, most of the bright protrusions of higher apparent height are absent, and smooth rod-like structures with a homogeneous apparent height of 1.6 Å (80% of the molecular species) coexist with species where one bright protrusion is still observed. Analogous experiments, where a sample containing *anti* α-bisdiketone heptacene precursors **1** is exposed to light with an identical wavelength as in case of **2** were realized, leading to the formation of heptacene (see Supplementary Fig. 11). We attribute this modification of the molecular structure to the gradual photodecarbonylation of the bridging α-diketone moieties as in the Strating-Zwanenburg reaction in solution^24,53^. This reaction has been previously studied in the context of the synthesis of acenes in polymethyl methacrylate (PMMA) and argon matrices which stabilized the formed acenes^26–28^. Light-induced formation of nonacene or heptacene in solution is expected to be hampered due to the formation of oxidized and/or dimerized species (see Supplementary Movie 1 that shows illumination of **2** in solution). It must be emphasized that photochemical decarbonylation of bridged α-diketone groups on metal surfaces may differ from the one realized in solution. In the former case two excitation processes are possible: a direct process, also present in photochemical reactions accomplished in solution, where photons excite the frontier electronic states of the molecules adsorbed on the metal substrate and induce a chemical reaction; and an indirect process where photons are absorbed by the substrate and generate hot charge carriers which enter the unoccupied states of the molecules via an inelastic scattering process. Experiments realized on a Au(111) surface with **2** using visible light of longer wavelength (*λ*_2_ = 627 nm, *ϕ*_photon_ = 3.2 × 10^18^ cm^−2^ s^−1^, *t* = 2.5 h) reveal a substantially low yield of only 6.5% of nonacene which is significantly lower than the 67% yield found upon illumination with *λ*_1_ = 470 nm. This possibly suggests that the light-induced decarbonylation of bridged α-diketone groups on Au(111) is governed by an indirect mechanism (we note that a photothermal effect can be excluded, as shown in Supplementary Fig. 12), though further mechanistic insight is out of the scope of this work and will be the goal of future investigations.

**Fig. 3:**
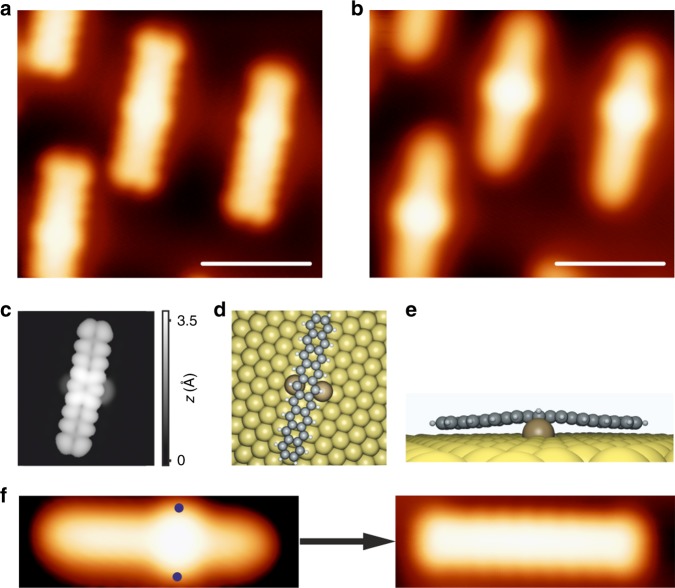
Au-nonacene interactions. **a**, **b** High-resolution STM images showing the Au-nonacene interaction as two protrusions at certain *V*_bias_. These interactions are mostly observed around the central benzene ring, though they can also be detected around neighboring benzene rings. **a** *V*_b_ = −1.2 V, *I* = 15 pA. Scale bar: 2 nm. **b** *V*_b_ = 2.3 V, *I* = 4 pA. Scale bar: 2 nm. **c** DFT simulated STM image of an individual nonacene molecule bound to two Au adatoms. *V*_b_ = −1.5 V. **d**, **e** Top and side views of the corresponding DFT equilibrium geometry. **f** High-resolution STM images demonstrating the removal of the Au adatoms. Left STM image *V*_b_ = 2.0 V, *I* = 65 pA. Right STM image *V*_b_ = −1.0 V, *I* = 50 pA

It is noteworthy that after photoconversion, lateral protrusions (especially noticeable at *V* > + 2 *V*) are discerned close to each nonacene molecule (Fig. 3a,b). We assign these protrusions to Au adatoms bound to the nonacene backbone. Importantly, after applying an alternative, thermal approach wherein the sample is annealed to 160–190 °C rather than exposed to light, lateral protrusions close to nonacene molecules are also observed at certain bias voltages *V*_bias_ (see supplementary Fig. 13). Figure 3c-e show the DFT simulated STM image and the corresponding top and side views of the DFT equilibrium geometry of an individual nonacene molecule interacting with two Au adatoms, which fully reproduces the main features of the experimental data (see Supplementary Fig. 14 for the nc-AFM image of a nonacene molecule bound to Au adatoms). Also, we note that DFT calculations reveal an energy gain of 0.17 eV for nonacene-Au in the configuration of Fig. 3d,e compared to a configuration with nonacene far away from two Au adatoms on the surface. The Au-nonacene interaction, with the Au atoms mostly observed at the center of the molecules, can be correlated with the well-known rise in reactivity of acenes with increasing length, with the central ring(s) being the most reactive. In line with this prediction and as an experimental manifestation of increasing reactivity of larger acenes, Zugermeier et al. have reported a stronger interaction of the central part of heptacene with Ag(111) leading to a slight but observable non-planarity of the heptacene backbone^54^. Most importantly, the mentioned Au-nonacene interactions can thus be considered as an indication of an increased open-shell character of nonacene molecules. Au-acene interactions have also been observed for heptacene molecules, albeit less frequently (only ~60% of the molecular species are bound to Au adatoms compared to ~95% for nonacene, see Supplementary Fig. 15). No indication of such interactions was observed in previous studies where shorter acenes were studied on the Au(111) substrate, further lending credibility to the prediction that the open-shell character of acenes significantly increases with their length. Finally, we note that these interactions can be disrupted by the following methodology: we locate the tip on one of the protrusions (marked by the blue dots in Fig. 3f) and turn off the feed-back loop. Subsequently, the tunneling voltage *V*_b_ is increased to ~ 2.5 V for a few seconds, inducing the displacement of the Au adatoms, which then allows the structural and electronic characterization of pristine nonacene species on the metal substrate.

At this point a comment regarding the role of the Kekulé and the non-Kekulé structures in the radical character of acenes is appropriate. The closed-shell Kekulé structure of nonacene can be represented with one migrating aromatic π-sextet (see Supplementary Fig. 16 (I)); however, one could also construct a non-Kekulé open-shell structure (see Supplementary Fig. 16 (II)) wherein an extra stabilizing aromatic π-sextet is accommodated in the chemical structure upon formal loss of a π-bond and associated unpaired electrons. This non-Kekulé structure is consistent with the observed experimental picture where the increased unpaired electron density around the central rings manifests itself in the interaction of nonacene with Au adatoms, which drives the system from a purely closed-shell structure towards an open-shell structure.

We have also investigated the tip-induced cleavage of α-diketone groups from **2**. For these experiments, the STM tip is placed at a fixed position above an α-diketone moiety of **2** and the feedback loop is turned off. The voltage is then ramped while monitoring the current between tip and sample. Reproducibly, a sudden decrease in the current signal at a threshold of −2.4 ± 0.2 V is detected, which we attribute to the cleaving of the α-diketone moiety, as is confirmed by a subsequent STM scan of the same area. Interestingly, the use of the same procedure employed for the cleavage of the second α-diketone moiety shows an increase in the threshold voltage to −3.5 ± 0.1 V (see Supplementary Fig. 17).

**Fig. 4:**
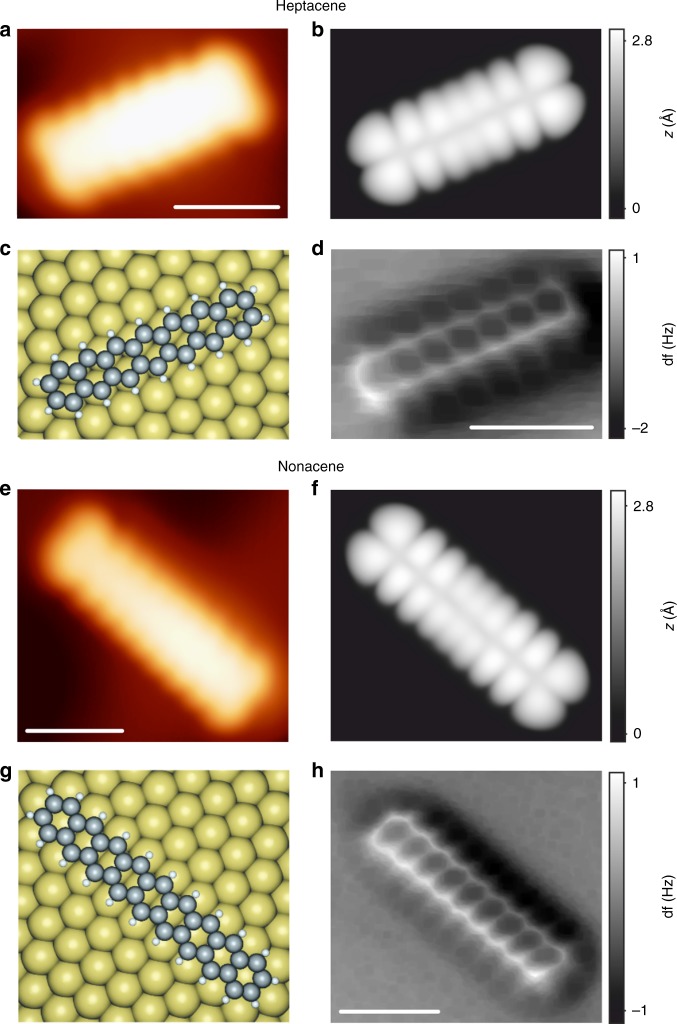
Heptacene and nonacene on Au(111) surface. **a**, **e** High-resolution STM images of heptacene and nonacene species, respectively. Nonacene was obtained by the procedure described in Fig. 2f. *V*_b_ = −1 V, *I* = 4 pA. **b**, **f** DFT simulated STM images at −1 V. **c**, **g** Top view of the DFT equilibrium geometries of heptacene and nonacene adsorbed on Au(111). **d**, **h** Constant-height frequency-shift nc-AFM images acquired with a CO-functionalized tip (z-offset 30 pm below STM set point at −5 mV, 10 pA). Scale bars 1 nm

Figure 4a, e depict constant-current STM images of individual heptacene and nonacene molecules, respectively, taken with a CO-functionalized tip, yielding enhanced resolution. The distinct appearance of heptacene (Fig. 4a) and nonacene (Fig. 4e), with seven and nine lobes along the long molecular axis, is in good agreement with the simulated STM images at the corresponding bias voltages (Fig. 4b, f, respectively). In Fig. 4c, g we show the DFT equilibrium geometries for the Au(111)-adsorbed heptacene and nonacene, respectively. The molecules adsorb in a planar configuration and 3.1 Å above the first Au(111) substrate layer, indicating weak physisorption. Ultimate structural details for both molecules are accessible by non-contact atomic force microscopy (nc-AFM) measurements using a CO-functionalized tip^37,54^. Figure 4d, h show the constant-height frequency-shift images where features assigned to seven and nine benzene rings directly confirm the obtained molecular structures as heptacene and nonacene. Moreover, the zigzag-edge carbon atoms in both molecules clearly have the expected single hydrogen termination, confirming the formation of pristine acenes.

### Electronic ground states of heptacene and nonacene

**Fig. 5:**
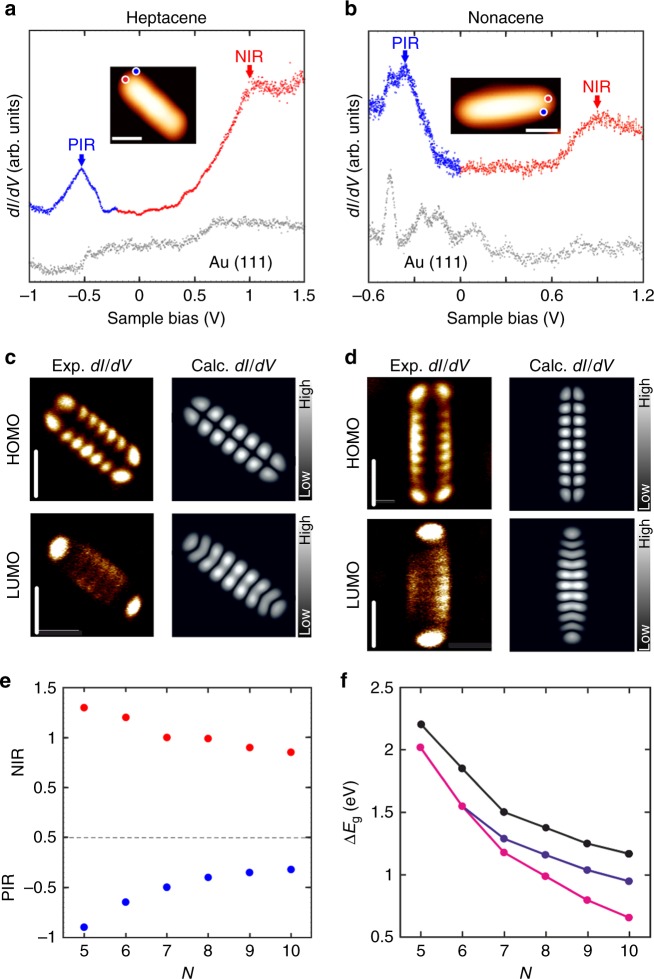
Electronic structure of heptacene and nonacene. **a**, **b** Differential conductance spectra of heptacene (**a**) and nonacene (**b**); the blue curves were acquired at the corner position while the red curves were acquired at the apex of the molecule, as indicated in the constant-current STM images (**a**: *V* = 1 V, *I* = 40 pA, **b**: *V* = 2 V, *I* = 5 pA) in the inset. **c**, **d** Experimental constant-current differential conductance maps at the PIR and the NIR (left), and DFT simulations of the corresponding HOMO for the N + 1 and N − 1 electron system in gas phase (right) of heptacene (**c**) and nonacene (**d**). Current values: 40 pA (**c**, top), 80 pA (**c**, bottom), 60 pA (**d**, top), 90 pA (**d**, bottom). **e** Experimentally determined energy positions of the PIR and the NIR, respectively for acenes from pentacene to decacene on Au(111). Data points for pentacene^36^, hexacene^35^, octacene^44^ and decacene^43^ are taken from literature. **f** Experimental electronic gaps (black curve) together with computed quasiparticle energy gaps for the singlet open-shell (blue curve) and closed-shell (pink curve) configurations, including screening effects from the Au(111) substrate. Scale bars: **a**, **b**, **c**: 1 nm, **d**: 0.5 nm. Prior to recording the dI/dV spectra, tip heights were stabilized with the following set points: −1 V, 40 pA (**a**, blue curve), 1.5 V, 40 pA (**a**, red curve), −0.6 V, 60 pA (**b**, blue curve) and 1.2 V, 50 pA (**b**, red curve)

To probe the electronic structure of the obtained heptacene and nonacene species, we have performed scanning tunneling spectroscopy measurements on individual molecules. For heptacene (Fig. 5a), differential conductance d*I*/d*V* spectra show strong peaks in the density of states at −0.5 V and +1 V, which we assign to the PIR and NIR deriving from the HOMO and LUMO, respectively. Importantly, in order to unambiguously probe the electronic structure of nonacene, electronic characterization reported herein has been performed on pristine nonacene species obtained after Au adatom removal from corresponding Au-nonacene entities. For nonacene (Fig. 5b), the position of HOMO and LUMO are found at −0.35 V and +0.9 V, respectively, which is in excellent agreement with the experimental HOMO-LUMO gap value for nonacene determined in a previous report by Zuzak et al.^42^. Hence, heptacene and nonacene present a HOMO-LUMO gap of 1.50 eV and 1.25 eV on Au(111), significantly less than the previously reported gap of 2.2 eV for pentacene^36^ and 1.85 eV for hexacene^35^ on Au(111) (Fig. 5e). Mapping of the d*I/*d*V* signal at the energetic positions of HOMO and LUMO for heptacene (Fig. 5c, left) and nonacene (Fig. 5d, left) allows us to visualize the spatial distribution of the corresponding molecular orbitals. The characteristic appearance of the HOMO with seven (heptacene) and nine (nonacene) lobes along the edges and a nodal plane along the molecular axis, and of the LUMO with the same number of lobes along the molecular axis matches well with the DFT-calculated local density of states (LDOS) maps of the HOMO of heptacene (Fig. 5c, right) and nonacene (Fig. 5d, right) in the gas phase, respectively, in the N + 1 and N − 1 electron configuration^55^. At this point, it is worth to comment on the nature of interaction between the acene molecules and the underlying surface, and the interpretation of STS results. Previous reports have observed molecular species with certain functionalities (e.g. sulfur) to strongly hybridize with underlying metal surfaces^50^, generating, for example, adsorption-induced density of states which may make a clear assignment of frontier orbital resonances challenging in STS experiments. However, in the present case, the excellent agreement between experimental d*I*/d*V* maps and the DFT-computed gas-phase molecular orbitals for both heptacene and nonacene affirms that the molecules are weakly physisorbed on the Au(111) surface without significant perturbation of molecular orbital resonances.

The inverse relation of electronic gap and acene length (visualized in Fig. 5e,f) can be rationalized in a simple way via Clar’s sextet rule for PAHs^56^. The combined effect of having only one stabilizing π-sextet, and the further weakening of aromaticity via sextet migration confers longer acenes with low electronic gaps and high chemical reactivity. This is in contrast to phenacenes, structural analogues of acenes with hexagonal rings fused in an armchair fashion, where there is an additive increase in the number of Clar sextets with increasing length and hence even longer members of the series show large electronic gaps and are stable compounds^57^. It is important to mention that in a recent study by Yeh and Chai^58^, it was pointed out that only energetic arguments (i.e. stabilization via additional aromatic sextets versus destabilization upon loss of one or more π–bonds) may not be sufficient to analyze the radical character of PAHs, but also the degeneracies of the non-Kekulé form must be taken into account. For larger acenes, the degeneracies of non-Kekulé structures with multiple stabilizing sextets and unpaired electrons rapidly increases, which suggests an increasing open-shell nature with increasing length.

In order to better understand the relation between the HOMO-LUMO gaps of acenes and their radical character, we have performed quasiparticle GW calculations for acenes including screening effects from the underlying Au(111) substrate (see method section for computational details). Two different electronic ground states from density functional theory have been employed in the GW calculations: First, a closed-shell (CS) ground state where the spatial orbitals for alpha (spin up) and beta (spin down) electrons are identical, and second, a singlet open-shell (SOS) ground state employing a single determinant where the spatial orbitals for alpha and beta electrons may differ. It is found that the SOS ground state and the CS ground state from DFT are identical for pentacene and hexacene, which results in matching CS and SOS GW gaps for *N* = 5 and *N* = 6 in Fig. 5f. For heptacene to decacene, the SOS ground state is found to be lower in energy than the CS ground state, with a larger GW gap for the SOS ground state.

For longer acenes, the CS GW gaps increasingly deviate from the experimental gaps with a maximum deviation of 0.5 eV for decacene. Figure 5f reveals that the SOS GW gaps are in much better agreement with the experimental spectroscopic values both in trend and absolute values. It can be debated whether SOS GW gaps can be related to experimental gaps since a multiconfigurational treatment including several Slater determinants is necessary for a proper treatment of SOS ground states. In principle, this multiconfigurational treatment is also necessary for computing the HOMO and LUMO maps in Fig. 5 c,d. A simplified but robust procedure is to employ the frontier orbital maps of the singly charged species from DFT^55^ which turn out to agree with the frontier orbitals of the neutral CS molecule from DFT^42^. On the one hand this finding suggests that the CS configuration still has a contribution to the multiconfigurational ground state which is clearly reasonable^59^. On the other hand, the deviation of CS GW gaps from the experimental ones, and the increased reactivity discussed earlier, clearly indicates that the higher acenes on an Au(111) surface acquire an open-shell character and cannot be described well by a CS configuration. We note that while the experimental determination of spin-polarization of larger acenes via spin-polarized STM would in principle be a conceivable way to visualize their electronic ground state, the extremely weak spin-orbit coupling in graphenic systems leads to very low magnetic anisotropies^60^ which precludes detection of spin polarization in such systems.

We envision that further combined experimental and theoretical investigations will unveil additional details on the nature of the ground state of large acenes. In this regard, advances in synthetic chemistry, in combination with the on-surface synthesis approach required to stabilize these species, will allow an in-depth characterization of acenes larger than nonacene, thus providing an appealing playground for the investigation of open-shell PAHs.

## Discussion

We have developed a previously unknown strategy towards the on-surface generation of large acenes, namely the visible-light-induced photodecarbonylation of α-bisdiketone precursors on single-crystalline metal substrates in an ultra-high vacuum (UHV) environment. Heptacene and nonacene precursors were deposited onto a Au(111) substrate and subsequently exposed to visible light (λ = 470 nm) which lead to the photochemical dissociation of the bridging *α*-diketone moieties and the formation of heptacene and nonacene. Scanning tunneling spectroscopy measurements yield HOMO-LUMO gaps of 1.5 eV for heptacene and 1.25 eV for nonacene on Au(111). A comparison to computed quasiparticle HOMO-LUMO gaps, together with the observed binding of Au adatoms to the central ring edges of heptacene (observed in ~60% of the molecules) and nonacene (observed in ~95% of the molecules), which is not observed for shorter acenes, demonstrate that acenes larger than hexacene possess an increasing open shell character on Au(111), which endows higher acenes with large reactivity. The results reported herein illustrate the generation and atomically-resolved structural and electronic characterization of surface-supported higher acenes, which have been elusive for many decades. We anticipate that this will stimulate further study of compounds of the acene series and their interesting ground states, and ultimately contribute to the development of organic materials for semiconductor applications.

## Methods

### Sample preparation and STM/nc-AFM measurements

Experiments were performed under UHV conditions (base pressure below 5 × 10^−10^ mbar) with two Scienta-Omicron scanning probe microscopes: a low-temperature STM and a low-temperature STM/AFM.

The Au(111) substrate was prepared by repeated cycles of Ar^+^ sputtering (*E* = 1 keV) and subsequent annealing to 750 K for 15 min. All STM images shown were taken in constant current mode with electrochemically etched tungsten tips at a sample temperature of 5 K. Scanning parameters are specified in each figure caption. Molecular precursors (**1** and **2**) were thermally deposited onto a clean Au(111) surface held at room temperature with a typical deposition rate of 0.4 Å min^−1^ (sublimation temperature ~610 K and 680 K respectively). Due to the high sublimation temperature of **2**, ~16% of the molecular precursor show a shorter length which is attributed to the partial decomposition upon sublimation (statistics out of 200 molecules). Once on the substrate, the intactly deposited precursors did not present any sign of decomposition/deprotection during their characterization which took a maximum of two days.

Nc AFM measurements were performed with a tungsten tip attached to a tuning fork sensor^54^. The tip was a posteriori functionalized by the controlled pick-up of a single CO molecule at the tip apex from the previously CO-dosed surface. The functionalized tip enables the imaging of the intramolecular structure of organic molecules^61^. The sensor was driven at its resonance frequency (22,350 Hz) with a constant amplitude of ~70 pm. The shift in the resonance frequency of the tuning fork (with the attached CO-functionalized tip) was recorded in constant-height mode (Omicron Matrix electronics and HF2Li PLL by Zurich Instruments). The STM and nc-AFM images were analyzed using WSxM.

### Light source details

After precursor deposition, the sample was exposed to visible light (Blue (470 nm) LUXEON Rebel LED, mounted on a 40 mm round base with seven LEDs, 490 lm @ 700 mA) at RT to achieve the heptacene (**1b**) and nonacene (**2b**) compounds, respectively. Subsequently, the sample was introduced in the STM and cooled down to 5 K for inspection. The light source was located outside the STM chamber at ~20 cm from the sample and with an illumination angle of 45° with respect to the surface. Exposure to longer wavelength light was done with a red (627 nm) LUXEON Rebel LED, mounted on a 40 mm round base with seven LEDs (525 lm @ 700 mA). Fermi-edge measurements of the Au(111) substrate were performed in a UHV system at a base pressure below 3 × 10^−11^ mbar using a UV-source with focusing mirror from Focus GmbH (HIS14) operated at HeI (21.208 eV). The data was acquired in normal emission configuration using a Scienta R3000 display analyzer operated at a pass energy of 5 eV in sweep mode with a step size of 1 meV. Light exposure (470 nm, Blue LUXEON Rebel LED mounted on a 40 mm round base with seven LEDs, 490 lm @ 700 mA) was realized under the same conditions as mentioned above for the sample after precursor deposition.

### Calculation methods

To obtain the equilibrium adsorption geometries within DFT, to simulate STM images and for the GW calculations, we used the CP2K code^62,63^ (see SI for full details). The surface/adsorbate systems were modeled within the repeated slab scheme^64^, i.e., a simulation cell contained 4 atomic layers of Au along the [111] direction and a layer of hydrogen atoms to passivate one side of the slab in order to suppress one of the two Au(111) surface states. Fouty angstrom of vacuum were included in the simulation cell to decouple the system from its periodic replicas in the direction perpendicular to the surface. We employed eigenvalue-self consistent GW for gas phase acenes^65^ and applied an image charge model^66^ to account for the screening by the metal surface.

The DFT HOMO maps of charge-neutral acenes in Fig. 5c,d were obtained from a calculation with a single positive charge, while for the LUMO maps of the charge-neutral acenes; the HOMO map of the singly negative charged species was used (see also Supplementary Fig. 18 for a full comparison). This procedure was adopted^55^ since it guarantees a ground state with less multiconfigurational character that is less problematic to treat with DFT. To obtain simulated STM images^67,68^, within the Tersoff-Hamann approximation^69,70^, we extrapolated the electronic orbitals to the vacuum region in order to correct the wrong decay in vacuum of the charge density due to the localized basis set^70^.

Full details on synthesis and computational details can be found in the Supplementary Information.

## Supplementary Information

Supplementary Information

Description of Additional Supplementary Files

Supplementary Movie 1

## Data Availability

The data that support the findings of this study are available from the corresponding authors upon reasonable request.
