# Supplementary material for: On-surface light-induced generation of higher acenes and elucidation of their open-shell character
Source: Nat Commun. 2019 Feb 20;10:861. doi: 10.1038/s41467-019-08650-y (PMC6382834; doi:10.1038/s41467-019-08650-y)
Supplement: Supplementary file 1 — Supplementary Information [file 41467_2019_8650_MOESM1_ESM.pdf]

On-surface light-induced generation of higher acenes and elucidation of their open-shell character

Urgel *et al*.

Supplementary Methods

1. Materials and Methods

1-1. Synthesis and characterization

Reagents for synthesis were purchased from Wako, Nacalai Tesque, and Sigma Aldrich, and were reagent-grade quality, obtained commercially, and used without further purification. For spectral measurements, spectral-grade solvents were purchased from Nacalai Tesque. Unless stated otherwise, column chromatography was carried out on silica gel 60N (Kanto Chemical, 40–50 μm). Analytical thin layer chromatography (TLC) was performed on Art. 5554 (Merck, KGaA). Melting points (m.p.) were measured with a YAMAKO MP-J3. IR spectra were recorded on a JASCO FP-6600 and are reported as wavenumbers ν in cm^{-1} with band intensities indicated as s (strong), m (medium), w (weak). ^{1}H NMR (300 MHz, 400 MHz and 600 MHz) and ^{13}C NMR (100 MHz and 150 MHz) spectra were recorded (as indicated) either on a JEOL JNM-AL 300, JEOL JNM-ECX 400 and JEOL JNM-ECX 600 spectrometer and are reported as chemical shifts (δ) in ppm relative to TMS (δ = 0). Broad peaks are marked as br. MS was performed on an APCI (Bruker micrOTOF II-KR). High resolution MS was performed on a MALDI-TOF (Bruker Autoflex II) or JEOL AccuTOF JMS-T100LC.

2. Abbreviations. Ar: Argon; AcOEt: Ethyl acetate; APCI: Atmospheric pressure chemical ionization; *n*-BuLi: *n*-butyllithium; DCM: Dichloromethane; DIPEA: *N*,*N*-Diisopropylethylamine; DMSO: Dimethyl sulfoxide; HRMS: High resolution mass spectroscopy; MALDI-TOF: Matrix assisted laser desorption ionization-time of flight; m.p.: Melting point; MeOH: Methanol; NMO: *N*-metylmorpholine *N*-oxide; rt: Room tempera-

ture; TFAA: Trifluoroacetic anhydride; THF: Tetrahydrofuran; TLC: Thin layer chromatography; TMS: Tetramethylsilane.

3. Synthesis

3-1. Synthesis of α-bisdiketone heptacene precursor (1)

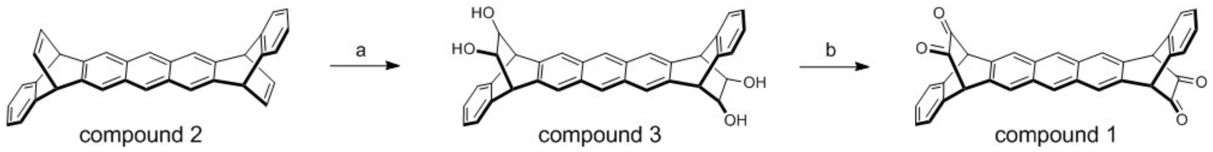

Supplementary Figure 1. a) OsO_4, NMO, acetone, rt, 5 days, 88%; b) DMSO, TFAA, DIPEA, DCM, –70 °C, 2 h, 63%.

Compound 2 was synthesized according to procedures reported previously.[1]

Compound 3. To a suspension of **2** (460 mg, 1.1 mmol) was added a solution of NMO (1205 mg, 10.3 mmol) and OsO_4 (47 mg, microcapsule 10% w/w, 0.018 mmol) in acetone (500 ml). The mixture was stirred for 5 days at rt. The reaction was quenched with sodium hydrosulfite, and the reaction mixture was stirred for 10 min. After removing acetone under reduced pressure, the organic phase was extracted with AcOEt, and then washed with water and brine, and then dried over Na_2SO_4. The solvent was removed under reduced pressure. The residue was purified by silica gel column chromatography (DCM/AcOEt/THF = 4:1:0.1, R_f = 0.4), and further purified by reprecipitation with THF and hexane to afford **3** as a white solid (483 mg, 0.97 mmol, 88%).; m.p.: >300 °C; IR (KBr): 3392 (s), 2945 (s), 1443 (s), 1071 (s), 1009 (s), 909 (s); ^{1}H NMR (400 MHz, DMSO-d_6): 8.35 (s, 2H), 7.92 (s, 2H), 7.85 (d, 2H), 7.39–7.35 (m, 2H), 7.32–7.29 (m, 2H), 7.16–7.10 (m, 4 H), 4.80 (s, br, 2H), 4.77(s, br, 2H), 4.46 (s, br, 2H), 4.44 (s, br, 2H), 4.04 (s, br, 2H), 3.95 (s, br, 2H); ^{13}C NMR (150 MHz, DMSO-d_6) (The minor peaks are occasionally observed alongside of the main peaks, which are presumably from the structural

isomers differing in the orientation of OH groups. Thus, the minor peaks are also listed.): 140.30, 139.31, 137.74, 137.31, 137.03, 130.40, 130.29, 130.20, 130.07, 126.44, 126.11, 125.77, 124.71, 124.59, 123.54, 122.08, 67.47, 66.68, 51.00, 50.95; HRMS (MALDI-TOF): *m/z*: calcd for $C_{34}H_{26}O_4$, 498.1826; found: 498.1834.

Compound 1. To a mixture of DCM (12 ml) and DMSO (4 ml) was added TFAA (0.8 ml, 5.7 mmol) at –70 °C under Ar atmosphere. After stirring for 1 h at –70 °C, a solution of **2** (54 mg, 0.11 mmol) in DCM (28 ml) and DMSO (15 ml) was added slowly to the prepared Swern reagent, and then the reaction mixture was stirred for 2 h at –70 °C. After the stirring, DIPEA (3.7 ml, 21.3 mmol) was added, and the reaction mixture was stirred for 1 h at –70 °C. The reaction was quenched by 3 M HCl, and the residue was extracted with DCM. The combined organic phase was washed with water and brine, and then dried over Na_2SO_4. The solvent was removed under reduced pressure. The residue was purified by silica gel column chromatography (DCM/AcOEt = 5:1, R_f = 0.4), and further purified by recrystallization with DCM and pentane to afford **1** (51 mg, 0.1 mmol, 63%) as a yellow solid. m.p.: >300 °C; IR (KBr): 1755 (s), 1733 (s), 1459 (m), 1241 (m), 1124 (m), 923 (m); ^{1}H NMR (600 MHz, $CDCl_3$): 8.43 (s, 2H), 8.08 (s, 4H), 7.52-7.50 (m, 4H), 7.42-7.40 (m, 4H), 5.20 (s, 4H); ^{13}C NMR (150 MHz, $CDCl_3$): 184.76, 134.88, 132.03, 131.97, 129.89, 126.90, 126.58, 125.60, 60.65; MS (APCI): *m/z*: calcd for $C_{34}H_{18}O_4$, 490.1205 $[M]^+$; found: 490.8989.

3-2. Synthesis of α-bisdiketone nonacene precursor (2)

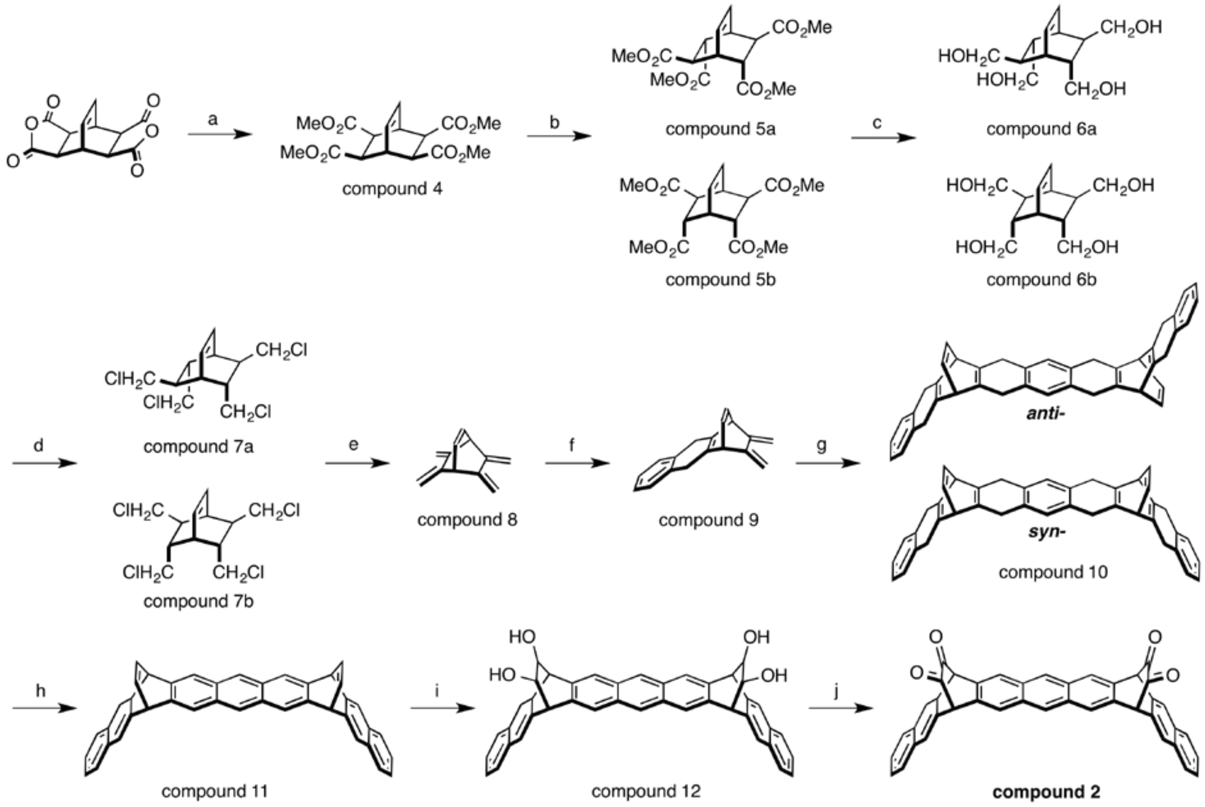

Supplementary Figure 2. a) Trimethyl orthoacetate, *p*-toluenesulfonic acid, MeOH, reflux, 4 days, 86%; b) Sodium methoxide, MeOH, reflux, 3 days, 13% (for **5a**), 32% (for **5b**); c) LAH, THF, reflux, 20 h, 85% (for **6a**), 89% (for **6b**); d) Thionyl chloride, pyridine, reflux, 2 h, 59% (for **7a**), 74% (for **7b**); e) Potassium *t*-butoxide, THF, reflux, 20 h, 78%; f) *n*-BuLi, 1,2-dibromobenzene, toluene, –10 °C – rt, overnight, 39%; g) *n*-BuLi, 1,2,4,5-tetrabromobenzene, toluene, –10 °C – rt, overnight, 54%; h) *p*-chloranil, K_2CO_3, toluene, reflux, 12 h, 45%; i) OsO_4, NMO, acetone, rt, 3 days, 39%; j) DMSO, TFAA, DIPEA, DCM, –65 °C, 3 h, 73%.

Compound 2 was basically synthesized according to procedures reported previously,[2] but some procedures were appropriately modified. The physical properties were identical with those reported in ref S2. Thus, only the synthetic procedure and ^{1}H NMR are shown below.

Compound 4. A suspension of bicyclo[2.2.2]oct-7-ene-2,3,5,6-tetracarboxylic dianhydride (37 g, 148 mmol) and *p*-toluenesulfonic acid (73 mg, 0.42 mmol) in *dry*-MeOH (59 ml) and trimethyl orthoacetate (56 ml) was heated to reflux for 4 days under Ar atmosphere. After the reaction, the solvent was removed under reduced pressure. The residue was dissolved in AcOEt, and the solution was washed with $NaHCO_3$-*aq*, water and brine. The combined organic phase was dried over Na_2SO_4, and the solvent was concentrated under reduced pressure. The precipitate was washed by diethyl ether to afford **4** (43.5 g, 128 mmol, 86%) as a white solid. ^{1}H NMR (300 MHz, $CDCl_3$): 6.44–6.40 (m, 2H), 3.62 (s, 12H), 3.58–3.33 (m, 2H), 3.11–3.06 (m, 4H).

Compound 5. A 28% MeOH solution of sodium methoxide (62 ml) was added to a MeOH solution (1000 ml) of **4** (129 g, 378 mmol), and was heated to reflux for 3 days under Ar atmosphere. Then, the reaction was quenched by addition of acetic acid (3.0 ml). The solvent was removed under reduced pressure, and the residue was dissolved in AcOEt, and the solution was run through short pad of silica, and the solvent was removed under reduced pressure. The resulting residue was washed with diethyl ether to afford **5b** (17 g, 50 mmol, 13%) as a white solid. Or, the resulting residue was filtrated off and the filtrate was concentrated to give **5a** as a viscous oil (42 g, 123 mmol, 32%). **5a**; ^{1}H NMR (300 MHz, $CDCl_3$): 6.35–6.29 (m, 2H), 3.77 (s, 6H), 3.68 (s, 6H), 3.52–3.48 (m, 2H), 3.20–3.17 (m, 2H), 2.98–2.95 (m, 2H). **5b**; ^{1}H NMR (300 MHz, $CDCl_3$): 6.57–6.51 (m, 1H), 6.12-6.07 (m, 1H), 3.69 (s, 6H), 3.66 (s, 6H), 3.58–3.53 (m, 2H), 3.29–3.27 (m, 2H), 2.90–2.87(m, 2H).

Compound 6a. LAH (11 g, 290 mmol) was added portionwise to a solution of **5a** (42 g, 123 mmol) in *dry*-THF (330 ml) at 0 °C under Ar atmosphere. Then, the reaction mixture was heated to reflux and was stirred for 20 h. After cooling to 0 °C, the reaction mixture was quenched by slowly addition of water (11 ml) and the reaction mixture was filtrated

off on short pad of silica, and the residue was washed with hot-ethanol. The combined filtrate was concentrated under reduced pressure, and the included water was removed by azeotropic distillation with toluene to afford **6a** (24 g, 105 mmol, 85%) as a white solid. ^{1}H NMR (300 MHz, D_2O): 6.19–6.13 (m, 2H), 3.54–3.42 (m, 4H), 3.23–3.06 (m, 4H), 2.52–2.45 (m, 2H), 1.39–1.32 (m, 2H), 1.06–0.99 (m, 2H).

Compound 6b. LAH (10 g, 264 mmol) was added portionwise to a solution of **5b** (24.5 g, 72 mmol) in *dry*-THF (540 ml) at 0 °C under Ar atmosphere. Then, the reaction mixture was heated to reflux and was stirred for 20 h. After cooling to 0 °C, the reaction mixture was quenched by addition of water (10 ml), and the reaction mixture was filtrated off on short pad of silica, and the residue was washed with hot-ethanol. The combined filtrate was concentrated under reduced pressure, and the including water was removed by azeotropic distillation with toluene to afford **6b** (15 g, 64 mmol, 89%) as a white solid. ^{1}H NMR (300 MHz, D_2O): 6.41–6.36 (m, 1H), 6.17–6.15 (m, 1H), 3.63–3.44 (m, 4H), 3.23–3.06 (m, 4H), 2.61–2.52 (m, 2H), 1.43–1.29 (m, 2H), 1.04–0.97 (m, 2H).

Compound 7a. Pyridine (28 ml) was added to thionyl chloride (33 ml) at 0 °C under Ar atmosphere. After stirring for 10 min, **6a** (24 g, 105 mmol) was added portionwise to the reaction mixture. Additional thionyl chloride (46 ml) was slowly added, and the reaction mixture was stirred for 30 min at 0 °C. The mixture was slowly heated to reflux for 2 h. After cooling to 0 °C, the solution was diluted with DCM (300 ml), and poured into ice water. The reaction mixture was extracted with DCM, and the combined organic phase was washed with $NaHCO_3$-*aq*, 3 M HCl and brine. The organic phase was dried over Na_2SO_4, and was concentrated under reduced pressure. The residue was washed with MeOH to afford **7a** (19 g, 62 mmol, 59%) as a white solid. ^{1}H NMR (400 MHz, $CDCl_3$): 6.37–6.34 (m, 2H), 3.79–3.75 (m, 2H), 3.51–3.46 (m, 2H), 3.40–3.35 (m, 2H), 3.31–3.26 (s, 2H), 2.99–

2.96 (m, 2H), 1.75–1.69 (m, 2H), 1.55–1.49 (m, 2H).

Compound 7b. Pyridine (16 ml) was added to thionyl chloride (20 ml) at 0 °C under Ar atmosphere. After stirring for 10 min, **6a** (14 g, 61 mmol) was added portionwise to the reaction mixture. Additional thionyl chloride (27 ml) was slowly added, and the reaction mixture was stirred for 30 min at 0 °C. The mixture was slowly heated to reflux for 2 h. After cooling to 0 °C, the solution was diluted with DCM (200 ml) and poured into ice water. The reaction mixture was extracted with DCM, and the combined organic phase was washed with $NaHCO_3$-*aq*, 3 M HCl and brine. The organic phase was dried over Na_2SO_4, and was concentrated under reduced pressure. The residue was washed with MeOH to afford **7b** (14 g, 45 mmol, 74%) as a white solid. ^{1}H NMR (300 MHz, $CDCl_3$): 6.60–6.55 (m, 1H), 6.07–6.02 (m, 1H), 3.85–3.79 (m, 2H), 3.60–3.45 (m, 4H), 3.26–3.20 (m, 2H), 3.14–3.02 (m, 2H), 1.76–1.69 (m, 2H), 1.52–1.45 (m, 2H).

Compound 8. Potassium *t*-butoxide (25.0 g, 222 mmol) was added to a THF solution (200 ml) of **7a** (5.0 g, 17 mmol) under Ar atmosphere. Then, the reaction mixture was heated to reflux for 20 h. After cooling to 0 °C, the reaction was quenched by addition of 2 M HCl, and the solvent was concentrated under reduced pressure. The residue was extracted with hexane, and the combined organic phase was washed with water, brine, and then dried over Na_2SO_4. The solvent was removed under reduced pressure, and the residue was purified by silica gel column chromatography (DCM/hexane = 1:1, R_f = 0.53 with hexane) to afford **8** (2.0 g, 13 mmol, 78%) as a white solid. ^{1}H NMR (300 MHz, $CDCl_3$): 6.39–6.35 (m, 2H), 5.23 (s, 4H), 4.93 (s, 2H), 3.86–3.84 (m, 4 H).

Compound 9. A toluene (75 ml) solution of *n*-BuLi (32.0 ml, 1.6 M in hexane) was added dropwise to a toluene solution (110 ml) of **8** (2.4 g, 15.4 mmol) and 1,2-dibromobenzene

(1.7 ml, 15.4 mmol) at –10 °C over 40 min under Ar atmosphere. The reaction mixture was stirred at –10 °C for 2 h and then stirred at rt overnight. Then, the reaction was quenched by adding MeOH (0.5 ml) and concentrated under reduced pressure. The residue was purified by silica gel column chromatography (hexane/DCM = 10:1, R_f = 0.25 with hexane) to afford **9** as a white solid (1.4 g, 5.9 mmol, 39%). ^{1}H NMR (300 MHz, $CDCl_3$): 6.39–6.35 (m, 2H), 5.23 (s, 4H), 4.93 (s, 2H), 3.86–3.84 (m, 4 H).

Compound 10. A toluene (60 ml) solution of *n*-BuLi (5.6 ml, 1.6 M in hexane) was added dropwise to a solution of **9** (1.4 g, 5.9 mmol) and 1,2,4,5-tetrabromobenzene (1.2 g, 3.0 mmol) in *dry*-toluene (90.0 ml) at –10 °C over 15 min under Ar atmosphere. Then, the reaction mixture was stirred overnight at rt. The reaction was quenched by addition of MeOH (0.5 ml), and the solvent was removed under reduced pressure. The residue was purified by silica gel column chromatography (DCM/hexane = 1:2, R_f = 0.5) to afford **10** (870 mg, 1.6 mmol, 54%) as a white solid. ^{1}H NMR (300 MHz, $CDCl_3$): 7.09 (s, 8H), 6.84–6.82 (m, 4H), 4.29–4.25 (m, 4H), 3.59 (s, 8H), 3.47 (s, 8H).

Compound 11. The suspension of **10** (597 mg, 1.62 mmol), *p*-chloranil (1.6 g, 6.59 mmol) and K_2CO_3 (3.1 g) in *dry*-toluene (300 ml) was heated to reflux for 12 h under Ar atmosphere. The hot-reaction mixture was filtered, and the filtrate was washed with hot-toluene. After cooling to rt, the filtrate was washed with 20% NaOH-*aq*, water, and then dried over Na_2SO_4. The organic phase was concentrated under reduced pressure, and the residue was dissolved in DCM, and *anti*-isomer was filtrated off. The filtrate was purified by silica gel column chromatography (hexane/DCM = 4:3, R_f = 0.6), and then further purified by recrystallization with DCM and MeOH to afford **11** (263 mg, 0.50 mmol, 45%) as a white solid. ^{1}H NMR (400 MHz, $CDCl_3$): 8.09 (s, 2H), 7.77 (s, 4H), 7.72 (s, 4H), 7.70–7.67 (m, 4H), 7.36–7.33 (m, 4H), 7.04–7.02 (m, 4H), 5.30–5.23 (m, 4H).

Compound 12. OsO_4 (270 mg, in microcapsule 10% w/w, 0.1 mmol) was added portion-wise to a suspension of **11** (615 mg, 1.2 mmol), NMO (1.5 g, 12.8 mmol) in acetone (100 ml). The mixture was stirred for 3 days at rt. Then, the reaction was quenched by addition of an aqueous solution of sodium hydrosulfite, and then the mixture was stirred for 10 min. After concentrated *in vacuo*, the brown residue was dissolved in AcOEt, and then was washed with water, brine, and then dried over Na_2SO_4. The organic phase was concentrated under reduced pressure, and the residue was purified by silica gel column chromatography (AcOEt/DCM/THF = 6:2:1, R_f = 0.43 with AcOEt/DCM = 2:1), and further purified by re-precipitation with THF and hexane to afford **12** (272 mg, 0.45 mmol, 39%) as a white solid. ^{1}H NMR (400 MHz, $CDCl_3$): 8.39 (s, 2H), 7.99–7.80 (m, 12H), 7.43–7.39 (m, 4 H), 4.86–4.79 (m, 4H), 4.59 (m, 4H), 4.12–4.02 (m, 4H).

Compound 2. TFAA (0.6 ml, 2.75 mmol) was added to a mixture of DCM (9.0 ml) and DMSO (3.0 ml) at –70 °C under Ar atmosphere. After stirring for 45 min, a solution of **11** (50 mg, 0.08 mmol) in DCM (33 ml) and DMSO (5.0 ml) was added slowly to the Swern reagent, and reaction mixture was stirred for 3 h at –65 °C. After the stirring, DIPEA (2.8 ml) was added and the reaction mixture was stirred for 2 h at –65 °C. The reaction was quenched by 1 M HCl (30 ml) and extracted with DCM. The combined organic phase was washed with water and brine, and dried over Na_2SO_4. The solvent was removed under reduced pressure. The residure was purified by silica gel column chromatography (AcO-Et/toluene = 2:1, R_f = 0.5), and further purified by recrystallization with DCM and pentane to afford **2** (18 mg, 0.03 mmol, 73%) as a yellow solid. ^{1}H NMR (400 MHz, $CDCl_3$): 8.43 (s, 2H), 8.10 (s, 4H), 7.96 (s, 4 H), 7.85–7.83 (m, 4 H), 7.54–7.7.49 (m, 4 H), 5.36 (s, 4 H).

4. NMR spectra

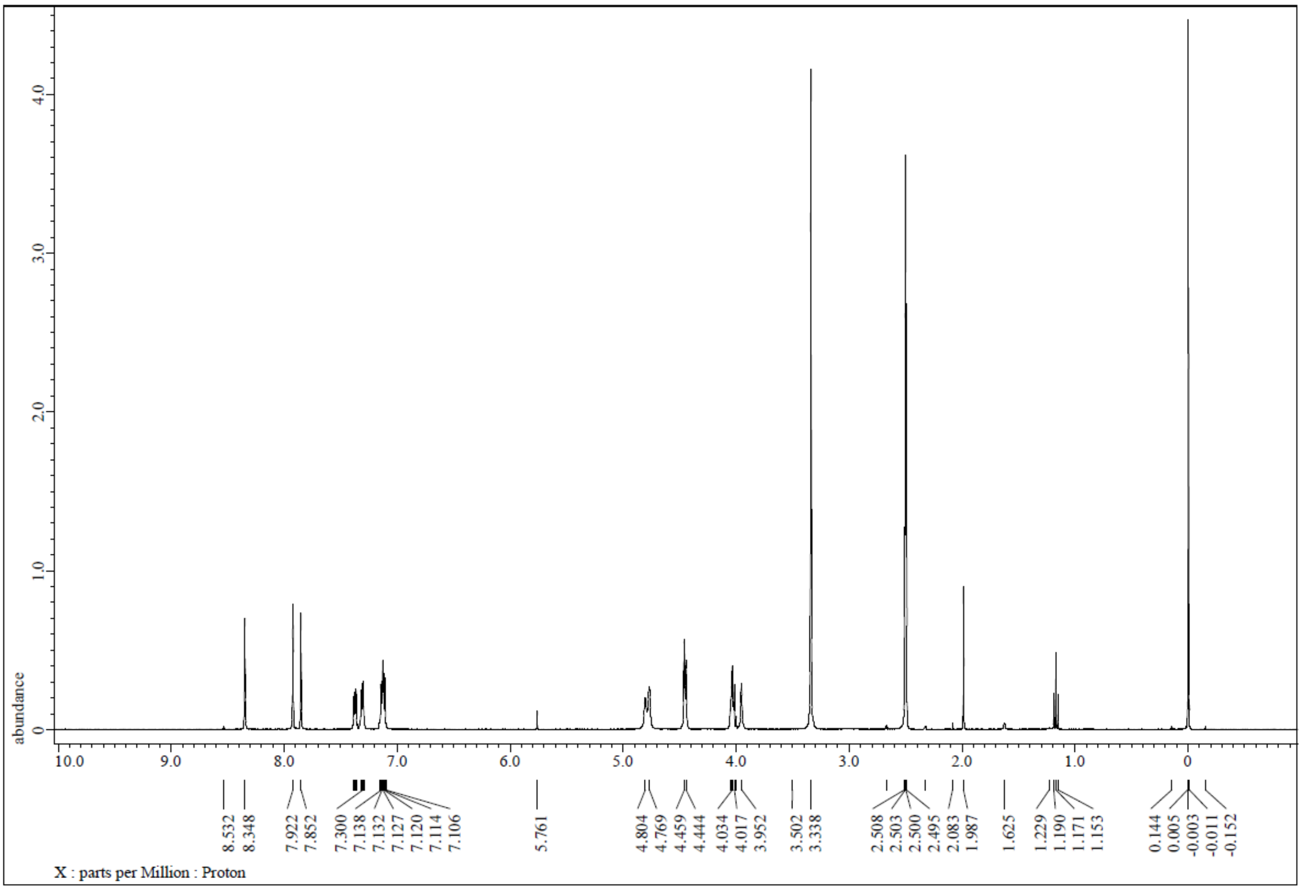

Supplementary Figure 3. ^{1}H NMR spectrum of **3** in DMSO-d_6.

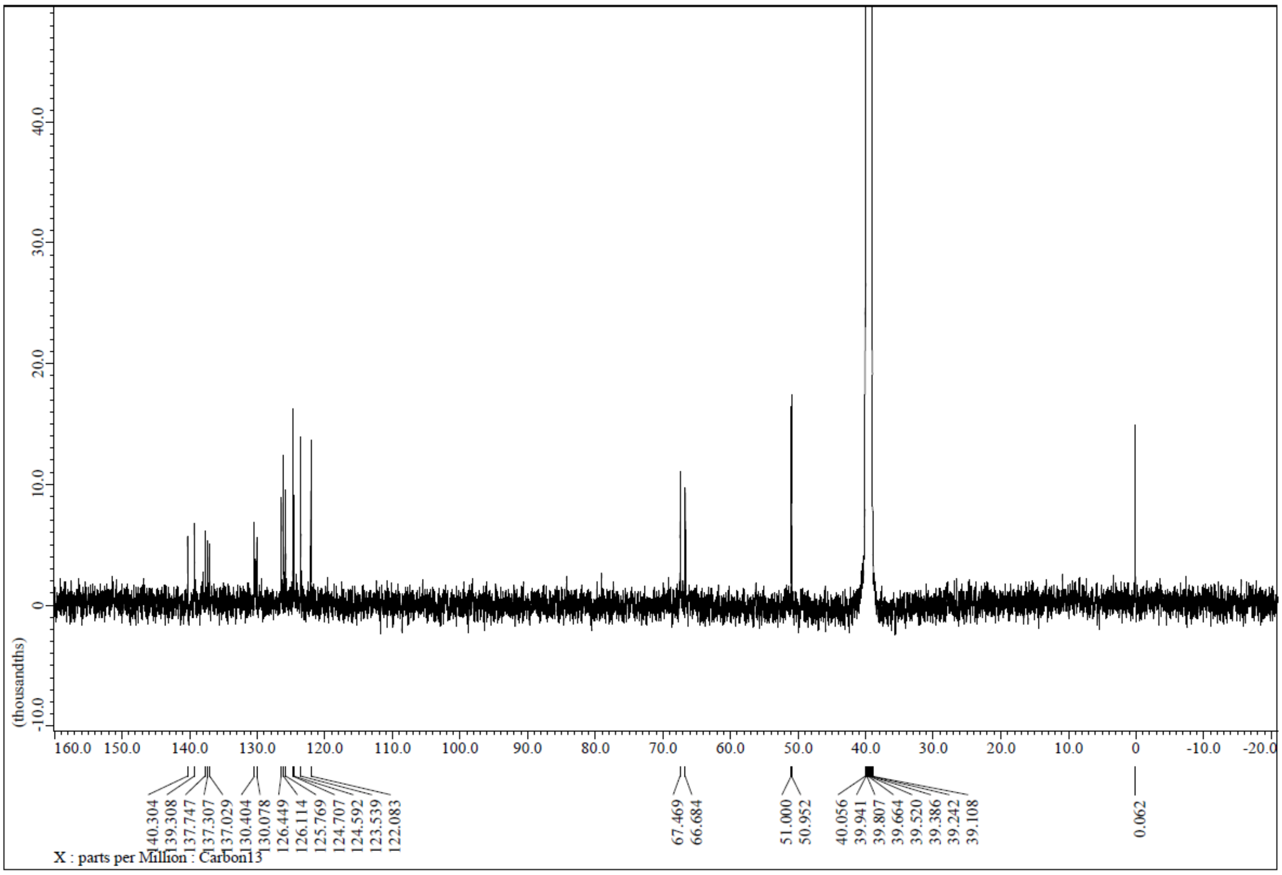

Supplementary Figure 4. ^{13}C NMR spectrum of **3** in DMSO-d_6 (Minor peaks are occasionally observed alongside of the main peaks, which are presumably from the structural isomers differing in the orientation of OH groups. The minor peaks are also listed in the spectroscopic characterization data above).

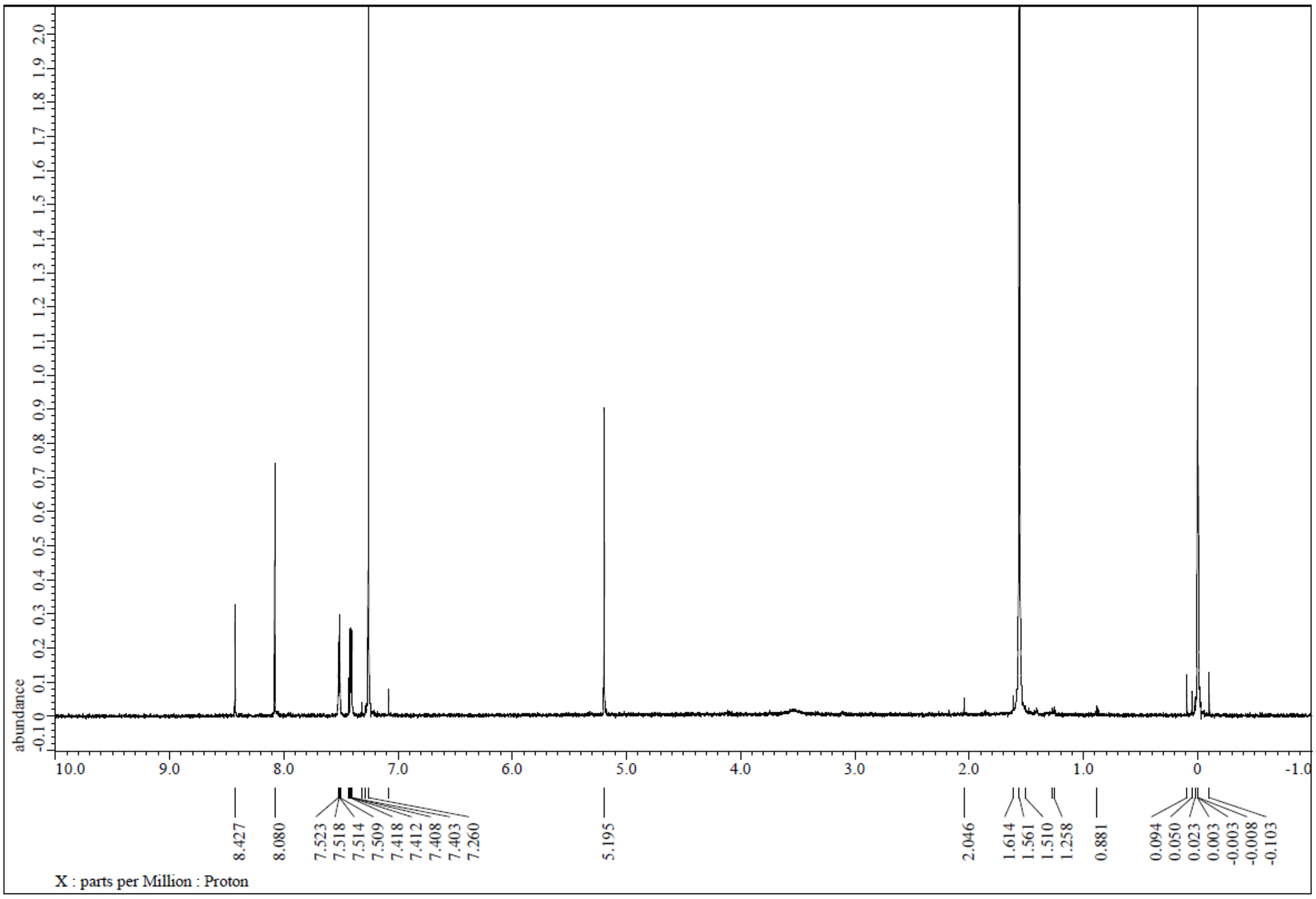

Supplementary Figure 5. ^{1}H NMR spectrum of **1** in $CDCl_3$.

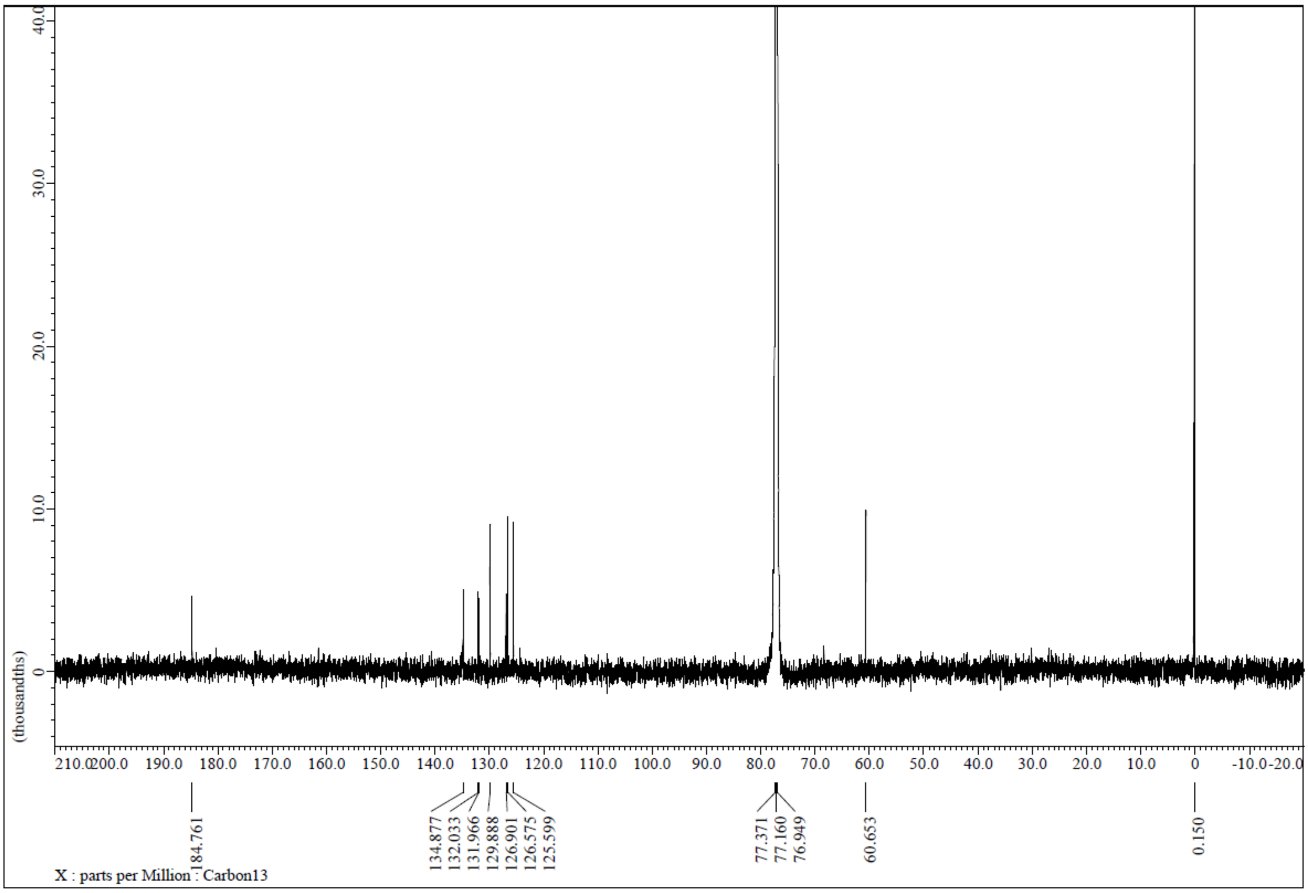

Supplementary Figure 6. ^{13}C NMR spectrum of **1** in $CDCl_3$.

5. HRMS

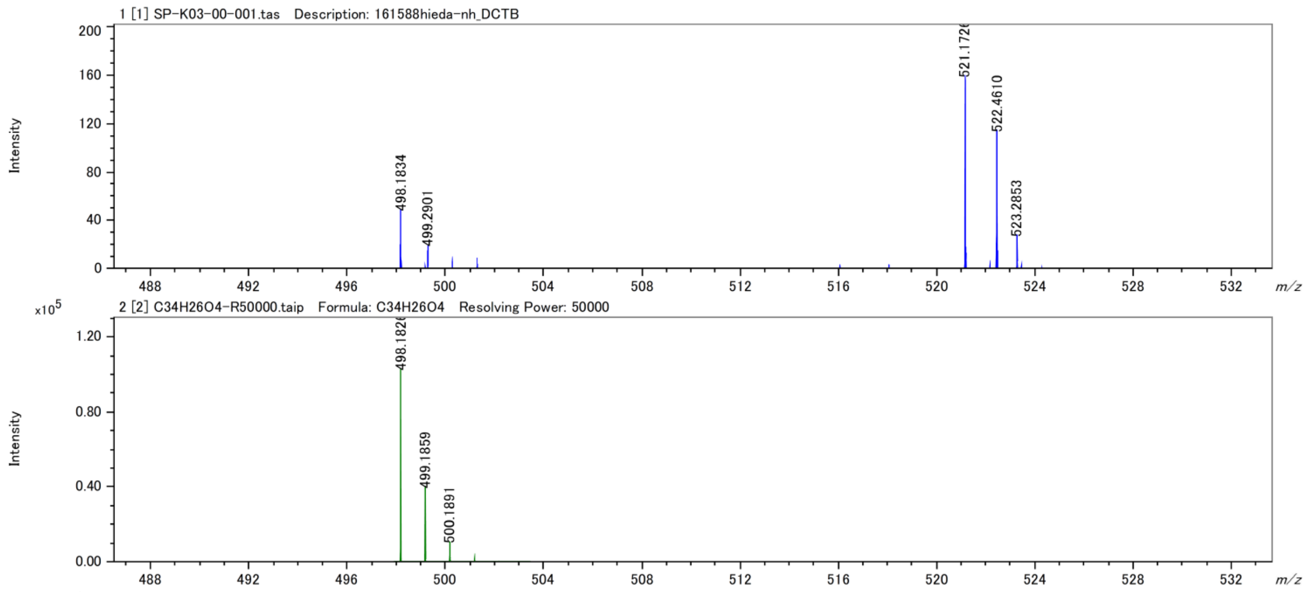

Elemental Composition Estimation

Parameters:

Mass	Tolerance	Electron Mode	Charge	DBE Range	Max Results
498.18343 ± 0.00249	5.0 ppm	Odd/Even	+1	−0.5 − 200.0	100

Elements

C 0 − 34 H 0 − 26 O 0 − 4 Na 0 − 1

Results:

#	Formula	Mass	DBE	Abs. Error (u)	Error (u)	Error (ppm)
1	C34 H26 O4	498.18256	22.0	0.00087	0.00087	1.74

Supplementary Figure 7. HR-MALDI-TOF mass spectrum of **3**.

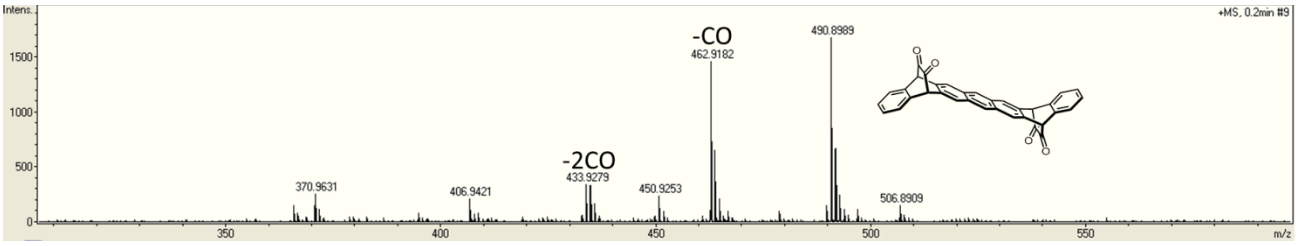

Supplementary Figure 8. APCI mass spectrum of **1**.

Supplementary Discussion

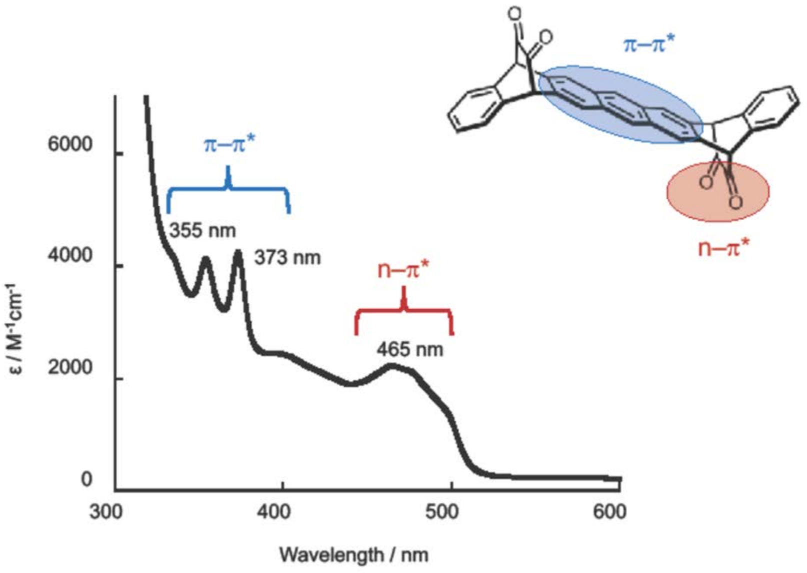

Supplementary Figure 9. UV-vis absorption spectrum of α-bisdiketone heptacene precursor in toluene.

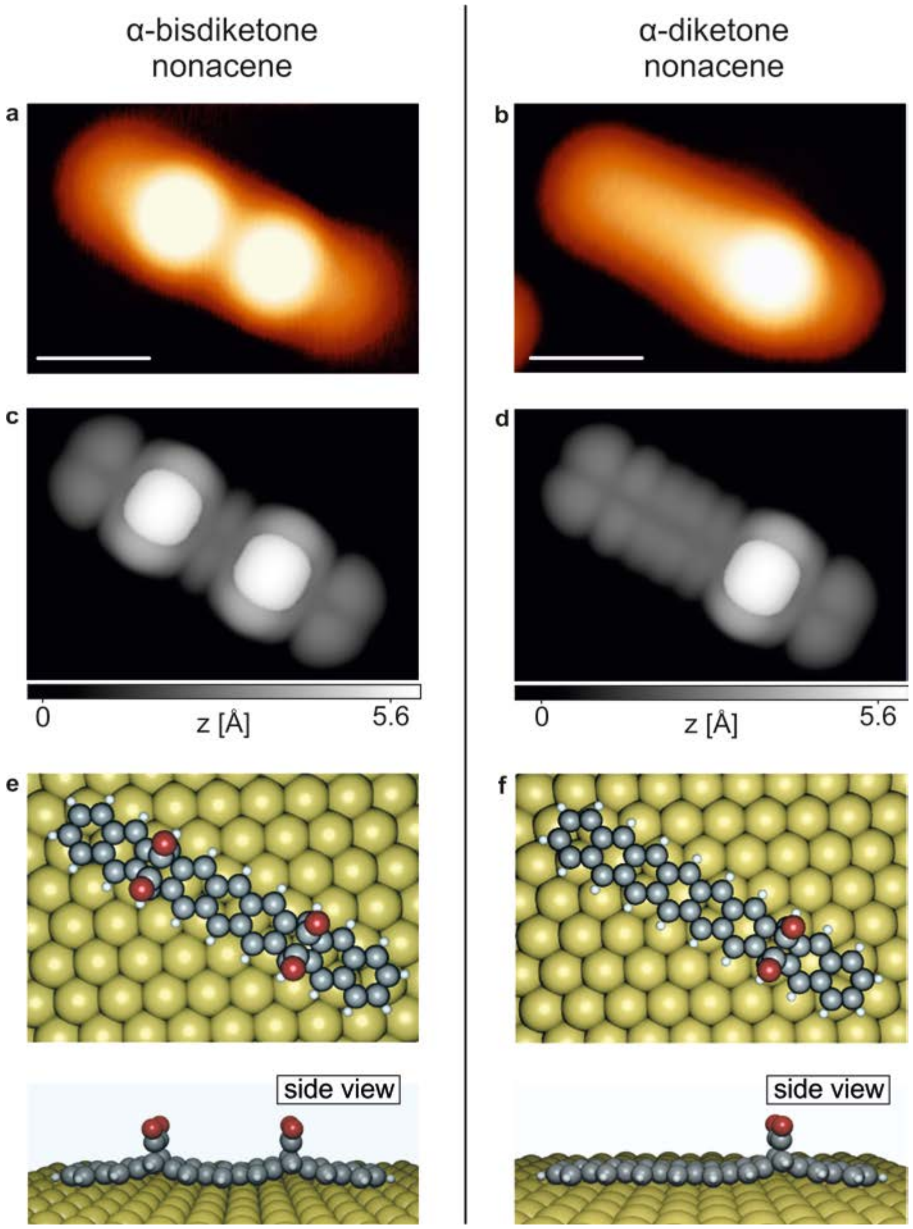

Supplementary Figure 10. α-bisdiketone and α-diketone nonacene species adsorbed on Au(111). (a,b) High-resolution STM image of α-bisdiketone nonacene and α-diketone nonacene on Au(111). V_b = - 1.5 V, I = 4 pA. Scale bar: 1 nm. (c,d) DFT simulated STM image of (a,b) respectively. V_b = -1.5 eV. (e,f) Top and side views of the corresponding DFT equilibrium geometries. DFT reveals adsorption and deformation energies of 3.24 eV and 0.53 eV for the α-bisdiketone nonacene and 3.77 eV and 0.28 eV for the α-diketone nonacene, respectively. A difference in adsorption height of ~ 1 Å between the central ring of the nonacene backbone or the molecular termini and the carbon atoms lifted up by the α-diketone groups is observed.

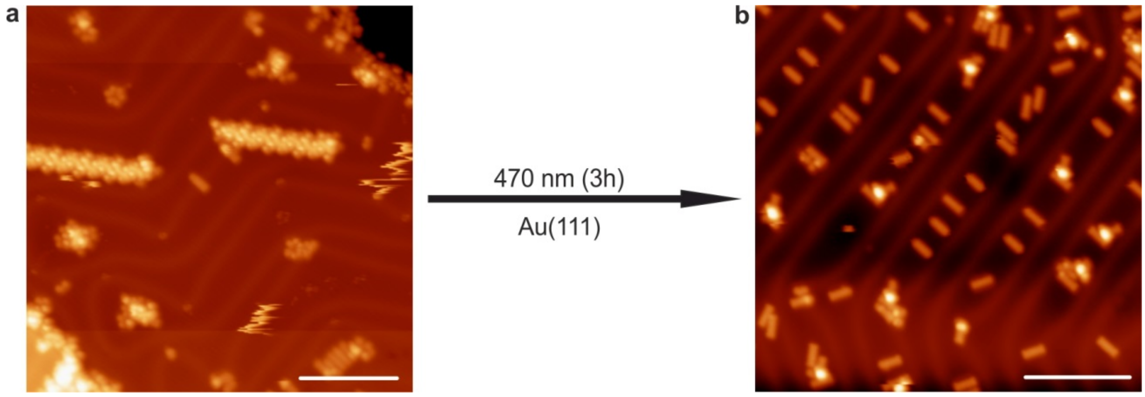

Supplementary Figure 11. Heptacene formation *via* photodissociation of α-bisdiketone heptacene precursors on the Au(111) surface. a) STM image of the room temperature deposition of the *anti*-conformational α-bisdiketone heptacene precursor on the substrate. V_b = - 1 V, I = 10 pA. b) STM image of heptacene species after light exposure (λ = 470 nm, 3h) of the heptacene precursor. V_b = -1 V, I = 7 pA. Scale bars: 10 nm.

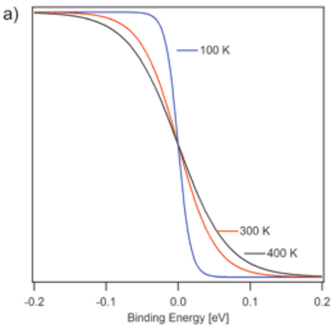

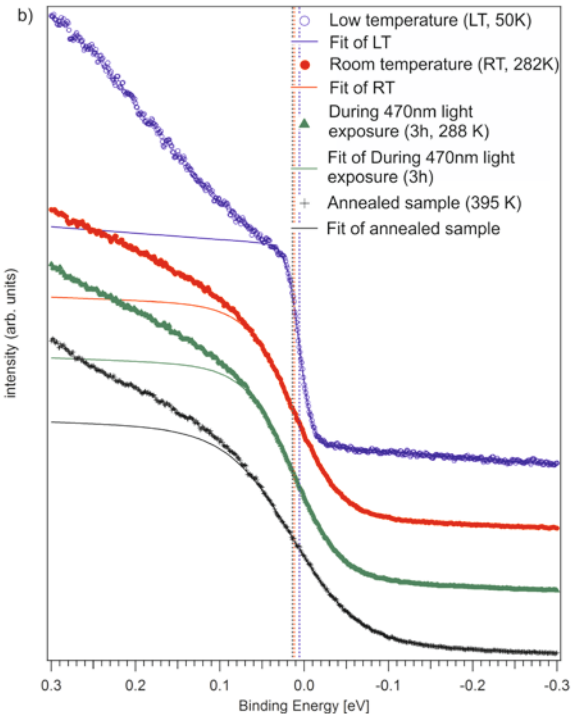

c)

	LT sample (50 K)	RT sample (282 K)	During 470nm light irradiation for 3h sample (288 K)	annealed sample (395 K)
Fermi energy (eV)	0.006	0.011	0.012	0.014
Temperature (K)	85	320	327	454

Supplementary Figure 12. a) Computed Fermi-Dirac distribution curves in blue, red and black for 100, 300 and 400 K, respectively, illustrating the temperature-dependent width of the Fermi edge. b) Experimental Fermi-Dirac distribution curves obtained by Ultraviolet Photoelectron Spectroscopy (UPS) measurements of the Au(111) substrate. Blue, red, green and black symbols correspond to measurements performed with the sample cooled to low temperature (LT, 50 K), without cooling or heating at room temperature (RT, 282 K), during exposure to 470 nm light for three hours at room temperature (3h, 288 K), and with the sample heated to 120 °C (395 K)), respectively. Temperatures indicated in brackets are those measured with a silicon diode located close to the sample holder, and are thus lower/higher than the actual sample temperature in case of sample cooling/heating, respectively. The curves are vertically offset for clear differentiation of each curve. Fitting of the experimental data in the range of -0.3 eV to +0.05 eV binding energy (black, green and red curves) or -0.3 eV to +0.04 eV binding energy (blue curve) has been done with the usual Fermi-Dirac function on a linear background. The resulting fits are shown as thin lines. c) Experimental values of the Fermi energy (chemical potential) and the sample surface temperature, determined from the fits shown in (b).

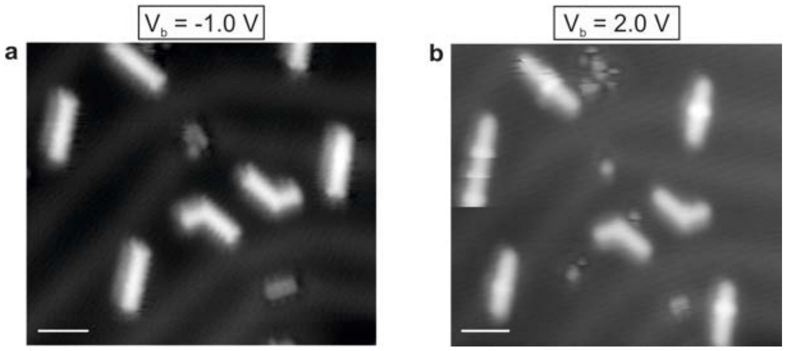

Supplementary Figure 13. Nonacene formation by thermal annealing of α-bisdiketone heptacene precursors on Au(111). a,b) STM images of nonacene molecules coexisting with some broken species after annealing the sample to 160 °C. At V_{bias} > + 2 V protrusions assigned to Au adatoms bound to the nonacene backbone are discerned. a) V_b = -1 V, I = 3 pA. b) V_b = 2 V, I = 2 pA. Scale bars: 2 nm.

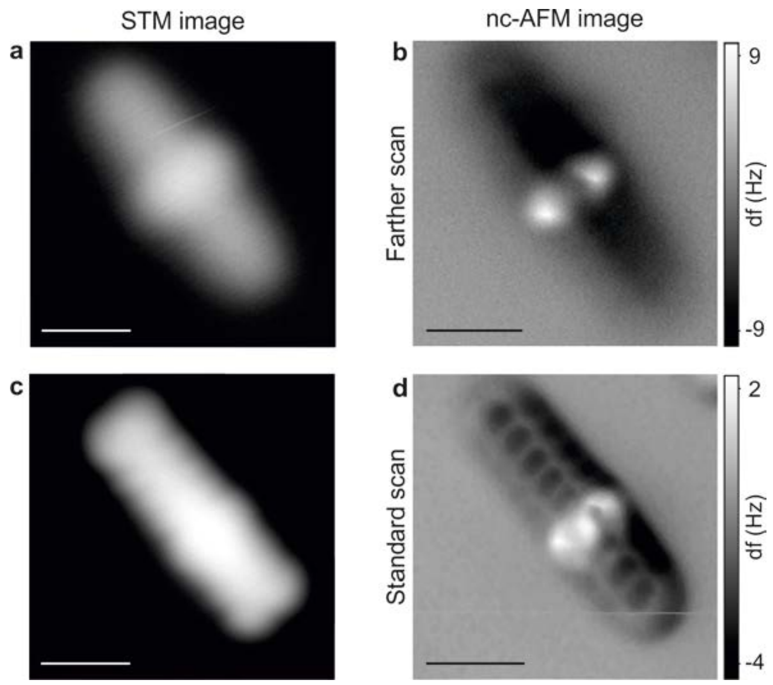

Supplementary Figure 14. STM and nc-AFM images of two nonacenes bound to gold adatoms. a,c) STM images of two nonacene molecules acquired with a CO-functionalized tip after light-induced photoconversion on Au(111). For both of them, two lateral protrusions appearing at different positions of the molecular backbone are recognized. a) V_b = 2 V, I = 5 pA. c) V_b = -1.5 V, I = 20 pA. Scale bars: 1 nm. b,d) Constant-height frequency-shift nc-AFM image of (a) and (c) respectively, acquired with a CO-functionalized tip. Scale bars = 1 nm. Two bright protrusions assigned to H atoms pointing upwards due to the molecule-Au adatom interaction are observed in accordance with the DFT equilibrium geometry model shown in Figure 2 d,e.

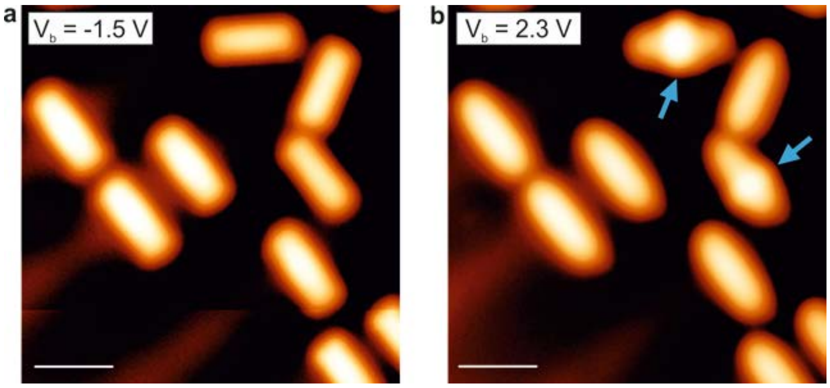

Supplementary Figure 15. Heptacene and Au-heptacene interactions. a,b) High-resolution STM images showing individual heptacenes together with some heptacene molecules bound to two Au adatoms. The Au-heptacene interactions can be clearly identified using tunneling voltages $V_b > +2$ V (b). Statistics out of 75 molecules reveal that ~ 60% of the molecular species are binding to Au adatoms. Blue arrows in (b) indicate heptacene molecules interacting with Au adatoms. a) $V_b = -1.5$ V, I = 2 pA. b) $V_b = 2.3$ V, I = 2 pA. Scale bars: 2 nm.

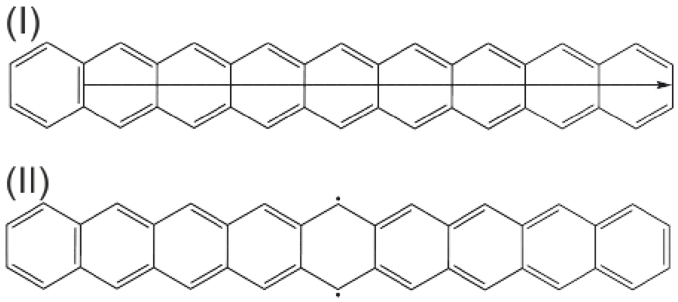

Supplementary Figure 16. Closed-shell Kekulé (top) and one of the many possible non-Kekulé (bottom) Clar structures of nonacene.

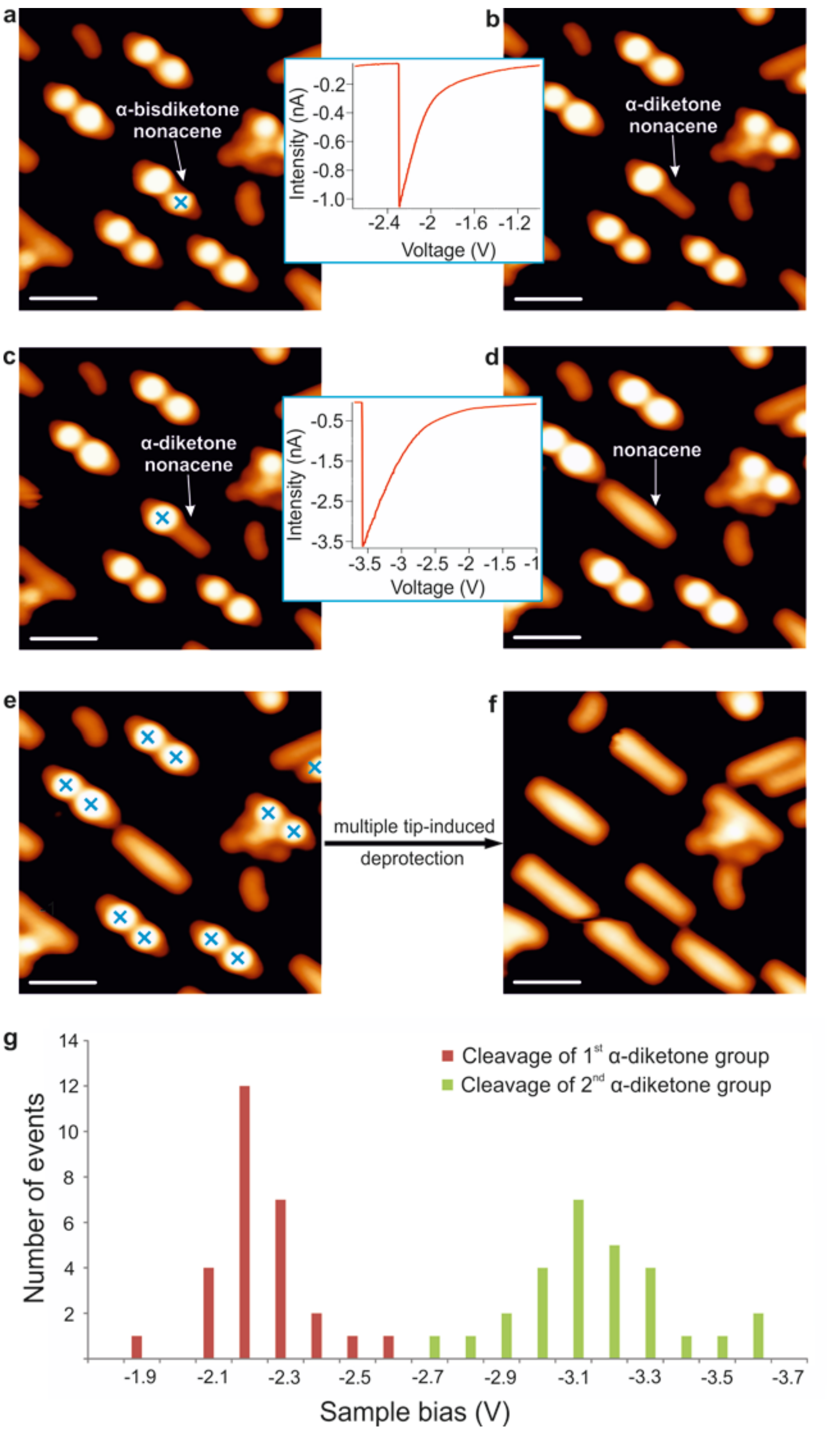

Supplementary Figure 17. Tip-induced deprotection of α-bisdiketone nonacene precursors on Au(111). a-d) STM topography images showing the cleavage of the α-diketone moieties. A voltage ramp of -1.0 to -3.0 V (a) and -1.0 to -3.7 V (c) is applied to the sample with feedback loop off. A sudden decrease in the current signal with a threshold at -2.3 V (graph in a,b) and -3.6 V (graph in c,d), attributed to the cleaving of α-diketone moieties, is observed and confirmed by a subsequent STM scan of the same area. e,f) STM topography images showing the formation of several nonacene molecules by tip-induced cleavage of the α-diketone moieties from nonacene precursors. Blue crosses indicate the position of the tip during the application

of the voltage ramp. a-d) V_b = -1.5 V, I = 3 pA. Scale bars: 2 nm. g) Histogram of the threshold voltage for the cleavage of α-bisdiketone nonacene precursors (statistics out of ~ 60 events). Similar results, i.e. sudden decrease in the current signal at voltages of 2.4±0.3 V and 3.3±0.3 V were detected after cleaving of the first and second α-diketone moiety, respectively.

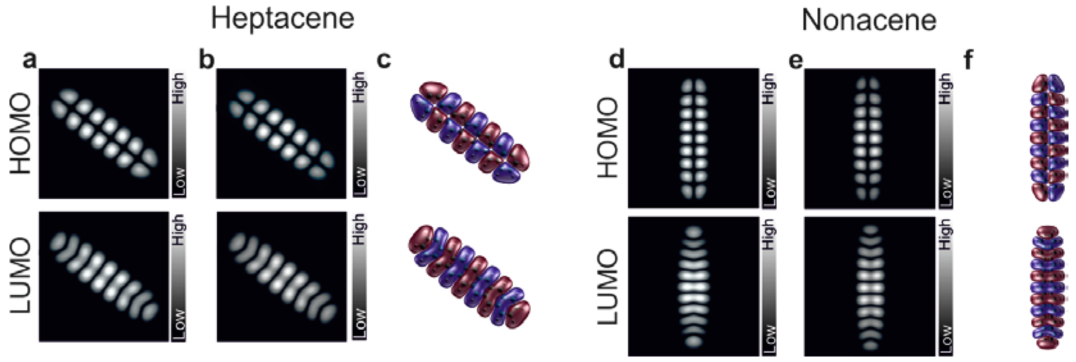

Supplementary Figure 18. Frontier orbitals of heptacene and nonacene. a,d) Constant height LDOS (plane 4 Å above molecular plane) for the HOMO of the N+1 charged system (top) and for the LUMO of the N-1 charged system (bottom) for heptacene and nonacene, respectively. b,e) Constant height LDOS (plane 4 Å above molecular plane) for HOMO (top) and LUMO (bottom) of the CS neutral system for heptacene and nonacene, respectively. c,f) isosurface of the probability amplitude (red and blue correspond to positive and negative isovalues) for HOMO (top) and LUMO (bottom) of the CS neutral system of heptacene and nonacene, respectively. The fact that the HOMO and LUMO of the CS neutral system are similar to the HOMO of the N+1 and N-1 charged systems respectively indicate that a CS character is still present in the ground state of the two systems.

Supplementary Notes

Computational details.

To obtain the equilibrium geometries of the molecules adsorbed on the Au(111) substrate and to compute corresponding STM images we used the CP2K code[3,4] implementing DFT within a mixed Gaussian plane waves approach.[5] The surface/adsorbate systems where modeled within the repeated slab scheme,[6] i.e., a simulation cell contained 4 atomic layers of Au along the [111] direction and a layer of hydrogen atoms to passivate one side of the slab in order to suppress one of the two Au(111) surface states. 40 Å of vacuum were included in the simulation cell to decouple the system from its periodic replicas in the direction perpendicular to the

surface. The electronic states were expanded with a TZV2P Gaussian basis set[7] for C and H species and a DZVP basis set for Au species. A cutoff of 600 Ry was used for the plane waves basis set. Norm-conserving Goedecker-Teter-Hutter[8] (GTH) pseudopotentials were used to represent the frozen core electrons of the atoms. We used the PBE parameterization for the general gradient approximation of the exchange correlation functional.[9] To account for van der Waals interactions we used the scheme proposed by Grimme.[10] We considered super-cells of 41.64 x 41.21 Å corresponding to 224 surface units. To obtain the equilibrium geometries we kept the atomic positions of the bottom two layers of the slab fixed to the ideal bulk positions, all other atoms were relaxed until forces were lower than 0.005 eV/Å.

For the GW calculations, we used the CP2K code[11]. We employed eigenvalue-self consistent GW[12,13] to compute the HOMO-LUMO gap of gas phase acenes based on PBE wavefunctions. We employed GTH pseudopotentials, the aug-DZVP basis from Ref. [11] and analytic continuation with a two-pole model. We apply an image charge model[14] to account for the screening by the metal surface. A distance of 1.58 Å between the image plane and the planar acene molecules has been used. This distance has been computed as difference between the adsorption height of 3.1 Å above the first substrate layer, see the main manuscript, and the distance of 1.42 Å between the image plane and the first substrate layer which has been taken from Ref. [15].

Supplementary References

1. Urgel, J. I. *et al.* On-Surface Synthesis of Heptacene Organometallic Complexes. *J. Am. Chem. Soc.* **139**, 11658–11661 (2017).

2. Tönshoff, C. & Bettinger, H. F. Photogeneration of Octacene and Nonacene. *Angew. Chem. Int. Ed.* **49,** 4125–4128 (2010).

3. *The CP2K developers group, CP2K is freely available. http://www.cp2k.org/.*

4. Hutter, J., Iannuzzi, M., Schiffmann, F. & VandeVondele, J. cp2k: atomistic simulations of condensed matter systems. *Wiley Interdiscip. Rev. Comput. Mol. Sci.* **4,** 15–25 (2014).

5. VandeVondele, J. *et al.* Quickstep: Fast and accurate density functional calculations using a mixed Gaussian and plane waves approach. *Comput. Phys. Commun.* **167,** 103–128 (2005).

6. Pickett, W. E. Pseudopotential methods in condensed matter applications. *Comput. Phys. Rep.* **9,** 115–197 (1989).

7. VandeVondele, J. & Hutter, J. Gaussian basis sets for accurate calculations on molecular systems in gas and condensed phases. *J. Chem. Phys.* **127,** 114105 (2007).

8. Goedecker, S., Teter, M. & Hutter, J. Separable dual-space Gaussian pseudopotentials. *Phys. Rev. B* **54,** 1703–1710 (1996).

9. Perdew, J. P., Burke, K. & Ernzerhof, M. Generalized Gradient Approximation Made Simple. *Phys. Rev. Lett.* **77,** 3865–3868 (1996).

10. Grimme, S., Antony, J., Ehrlich, S. & Krieg, H. A consistent and accurate ab initio parametrization of density functional dispersion correction (DFT-D) for the 94 elements H-Pu. *J. Chem. Phys.* **132,** 154104 (2010).

11. Wilhelm, J., Del Ben, M. & Hutter, J. GW in the Gaussian and Plane Waves Scheme with Application to Linear Acenes. *J. Chem. Theory Comput.* **12,** 3623–3635 (2016).

12. Blase, X., Attaccalite, C. & Olevano, V. First-principles GW calculations for fullerenes, porphyrins, phtalocyanine, and other molecules of interest for organic photovoltaic applications. *Phys. Rev. B* **83,** 115103 (2011).

13. Rangel, T., Hamed, S. M., Bruneval, F. & Neaton, J. B. Evaluating the GW Approximation with CCSD(T) for Charged Excitations Across the Oligoacenes. *J. Chem. Theory Comput.* **12,** 2834–2842 (2016).

14. Neaton, J. B., Hybertsen, M. S. & Louie, S. G. Renormalization of Molecular Electronic Levels at Metal-Molecule Interfaces. *Phys. Rev. Lett.* **97,** 216405 (2006).

15. Kharche, N. & Meunier, V. Width and Crystal Orientation Dependent Band Gap Renormalization in Substrate-Supported Graphene Nanoribbons. *J. Phys. Chem. Lett.* **7,** 1526–1533 (2016).
