# Supplementary material for: On-surface light-induced generation of higher acenes and elucidation of their open-shell character
Source: Nat Commun. 2019 Feb 20;10:861. doi: 10.1038/s41467-019-08650-y (PMC6382834; doi:10.1038/s41467-019-08650-y)
Supplement: Supplementary file 2 — Description of Additional Supplementary Files [file 41467_2019_8650_MOESM2_ESM.pdf]

Description of Additional Supplementary Files

File Name: Supplementary Movie 1
Description: Nonacene precursor light-illumination in solution
